# Supplementary material for: Low-nuclearity CuZn ensembles on ZnZrOx catalyze methanol synthesis from CO2
Source: Nat Commun. 2024 Apr 10;15:3101. doi: 10.1038/s41467-024-47447-6 (PMC11006684; doi:10.1038/s41467-024-47447-6)
Supplement: Supplementary file 1 — Supplementary information [file 41467_2024_47447_MOESM1_ESM.pdf]

Supplementary Information

Low-nuclearity CuZn ensembles on $ZnZrO_x$ catalyze methanol synthesis from CO_2

Thaylan Pinheiro Araújo,[1] Georgios Giannakakis,[1] Jordi Morales-Vidal,[2] Mikhail Agrachev,[3] Zaira Ruiz-Bernal,[4] Phil Preikschas,[1] Tangsheng Zou,[1] Frank Krumeich,[5] Patrik O. Willi,[1] Wendelin J. Stark,[1] Robert N. Grass,[1] Gunnar Jeschke,[3] Sharon Mitchell,[1] Núria López,[2] and Javier Pérez-Ramírez*[1]

[1] Institute of Chemical and Bioengineering, Department of Chemistry and Applied Biosciences, ETH Zurich, Vladimir-Prelog-Weg 1, 8093 Zurich, Switzerland

[2] Institute of Chemical Research of Catalonia (ICIQ-CERCA), The Barcelona Institute of Science and Technology, Av. Països Catalans 16, 43007 Tarragona, Spain

[3] Laboratory of Physical Chemistry, Department of Chemistry and Applied Biosciences, ETH Zurich, Vladimir-Prelog-Weg 2, 8093 Zurich, Switzerland

[4] Department of Inorganic Chemistry and Materials Institute (IUMA), Faculty of Sciences, University of Alicante, Ap. 99, E-03080 Alicante, Spain

[5] Laboratory of Inorganic Chemistry, Department of Chemistry and Applied Biosciences, ETH Zurich, Vladimir-Prelog-Weg 1, 8093 Zurich, Switzerland

*Corresponding author. E-mail: jpr@chem.ethz.ch

Table of Contents

1. Supplementary Methods 2
  1.1. Energy properties of the catalysts 2
2. Supplementary Discussion 3
  2.1. Modeling of the catalytic systems 3
    2.1.1. $ZnZrO_x$ 3
    2.1.2. Cu-ZrO_x 4
    2.1.2. Cu-$ZnZrO_x$ 4
  2.2. Additional insights into oxygen vacancy formation and dynamics 6
3. Supplementary Tables 7
4. Supplementary Figures 16
5. Supplementary References 46

1. Supplementary Methods

1.1. Energy properties of the catalysts

The potential energy (E) of several systems was computed using **Equations S1-S2** to rationalize (*i*) the tendency of Zn and Cu to be incorporated into m-ZrO_2 (−111) surface sites, (*ii*) the deposition of different amounts of Cu atoms on undoped and Zn-doped m-ZrO_2 surfaces, and (*iii*) the influence of oxygen vacancies. The formula units of the bulk of m-ZrO_2 polymorph ($E^{\mathrm{DFT}}_{\mathrm{ZrO_{2,bulk}}}$), molecular H_2 ($E^{\mathrm{DFT}}_{\mathrm{H_2,gas}}$), and H_2O ($E^{\mathrm{DFT}}_{\mathrm{H_2O,gas}}$) were used thermodynamic sinks. Bulk Zn ($E^{\mathrm{DFT}}_{\mathrm{Zn,bulk}}$) and Cu ($E^{\mathrm{DFT}}_{\mathrm{Cu,bulk}}$) metallic phases were employed as source of dopants. Zr_xO_y is the slab model of m-ZrO_2 (−111), which was represented with $p(1\times1)$ and $p(2\times2)$ expansions, where s and i indicate the number of Zr atoms in the m-ZrO_2 lattice substituted by Zn and Cu, respectively. Additionally, one oxygen vacancy was considered for the incorporation of each Zn and Cu atoms. Finally, n stands for the number of deposited Cu atoms.

$$\mathrm{Zr}_x\mathrm{O}_y + s\mathrm{Zn} + (i+n)\mathrm{Cu} + (\mathrm{s}+i)\mathrm{H}_2 \rightarrow \mathrm{Cu}_{i+n}\mathrm{Zn}_s\mathrm{Zr}_{x-s-i}\mathrm{O}_{y-s-i} + (s+i)\mathrm{ZrO}_{2,\mathrm{bulk}}\,(s+i)\mathrm{H}_2\mathrm{O} \quad \text{(S1)}$$

$$E = E^{\mathrm{DFT}}_{\mathrm{Zn}_s\mathrm{Cu}_{i+n}\mathrm{Zr}_{x-s-i}\mathrm{O}_{y-s-i}} + (s+i)E^{\mathrm{DFT}}_{\mathrm{ZrO}_{2,\mathrm{bulk}}} + (s+i)E^{\mathrm{DFT}}_{\mathrm{H_2O,gas}} - E^{\mathrm{DFT}}_{\mathrm{Zr}_x\mathrm{O}_y} - sE^{\mathrm{DFT}}_{\mathrm{Zn,bulk}} - (i+n)E^{\mathrm{DFT}}_{\mathrm{Cu,bulk}} \quad \text{(S2)}$$
$$-(s+i)E^{\mathrm{DFT}}_{\mathrm{H_2,gas}}$$

The formation energy (E_f) of ZnO and CuO was computed using **Equations S3-S4** to compare the oxygen affinity of metals (M = Zn and Cu). The energy references were the computed Zn and Cu bulks ($E^{\mathrm{DFT}}_{\mathrm{Zn,bulk}}$ and $E^{\mathrm{DFT}}_{\mathrm{Cu,bulk}}$, respectively) and molecular O_2 ($E^{\mathrm{DFT}}_{\mathrm{O_2,gas}}$).

$$M + \frac{1}{2}\mathrm{O}_2 \rightarrow M\mathrm{O} \quad \text{(S3)}$$

$$E_{\mathrm{f},M\mathrm{O}} = E^{\mathrm{DFT}}_{M\mathrm{O,bulk}} - E^{\mathrm{DFT}}_{M\mathrm{,bulk}} - \frac{1}{2}E^{\mathrm{DFT}}_{\mathrm{O_2,gas}} \quad \text{(S4)}$$

The tendency of a metallic Cu atom to alloy with Zn was assessed by means of the computed formation energy of Cu_5Zn_8 and CuZn obtained with **Equations S5-S6**.

$$x\mathrm{Cu} + y\mathrm{Zn} \rightarrow \mathrm{Cu}_x\mathrm{Zn}_y \quad \text{(S5)}$$

$$E_{\mathrm{Cu}_x\mathrm{Zn}_y} = (E^{\mathrm{DFT}}_{\mathrm{Cu}_x\mathrm{Zn}_y} - xE^{\mathrm{DFT}}_{\mathrm{Cu,\,bulk}} - yE^{\mathrm{DFT}}_{\mathrm{Zn,\,bulk}}) / x \quad \text{(S6)}$$

2. Supplementary Discussion

2.1. Modeling of the catalytic systems

Given the complexity of the different catalysts under study, particularly the multicomponent Cu-$ZnZrO_x$ system, we assessed over 200 different structures to select the most representative model for $ZnZrO_x$, Cu-ZrO_x, and Cu-$ZnZrO_x$. The selection was based on the DFT-computed energies to identify thermodynamically favored processes and on the compatibility with the in-depth experimental characterization.

XRD analysis showed that *t*-ZrO_2 partially transforms into *m*-ZrO_2 under CO_2 hydrogenation conditions in all the examined metal-promoted $ZnZrO_x$ catalysts (**Supplementary Fig. 3**). However, our previous UV-Raman spectroscopic studies of the $ZnZrO_x$ catalyst revealed that its surface, which is the most relevant substructure for catalytic purposes, fully transforms into the monoclinic ZrO_2 phase upon reaction.[1] Due to the lower metal promoter content (0.5 mol%) added to the $ZnZrO_x$ used in this study, it is likely that the surface of all metal-promoted $ZnZrO_x$ catalysts predominantly contains *m*-ZrO_2. Moreover, our previous DFT simulations indicated that both *t*-$ZnZrO_x$ and *m*-$ZnZrO_x$ surfaces selectively hydrogenate CO_2 to methanol, with the latter being slightly more active due to electronic and geometric effects.[1] Finally, other studies have shown that the monoclinic phase is thermodynamically favored under reaction conditions and is active for CO_2 hydrogenation to methanol.[2–5] Consequently, our modeling is based on slabs representing the *m*-ZrO_2 (–111), the most stable termination of this polymorph.[6,7]

2.1.1. $ZnZrO_x$

The incorporation of each Zn atom into surface sites of *m*-ZrO_2 is favored and stabilized by the formation of an oxygen vacancy ($ZnZrO_x$, $\Delta E = -0.04$ eV, **Fig. 5**), as previously reported.[1] Hence, this model was employed to describe the $ZnZrO_x$ catalyst since it is in line with the oxidic and isolated character of Zn sites identified by the EXAFS analysis under reaction conditions (**Fig. 4c**).

2.1.2. Cu-ZrO$_x$

We assessed the incorporation of Cu into several sites of *m*-ZrO_2(−111) with 0, 1, and 2 oxygen vacancies (50 configurations). The incorporation of Cu is endothermic (Cu@ZrO$_x$, ΔE = 1.86 eV, **Fig. 5**), but the formation of an oxygen vacancy also favors this process to a certain extent (**Supplementary Fig. 15**). The higher tendency of Zn to be incorporated compared to Cu is attributed to the higher oxidic character of the former, as indicated by the computed formation energies of their associated metal oxides ($E_{f,ZnO}$ = –2.89 eV and $E_{f,CuO}$ = –1.20 eV). Moreover, adsorption of a single Cu atom on the surface of *m*-ZrO_2 is also endothermic (Cu/ZrO_2, ΔE = 2.63 eV, **Fig. 5**) which explains the tendency of Cu sintering into nanoparticles upon reaction when supported on ZrO_2 (**Supplementary Fig. 14a**). Thus, the Cu-ZrO$_x$ system can be modeled as the two separated phases, *i.e.*, Cu(111) and *m*-ZrO_2(−111).

2.1.3. Cu-ZnZrO$_x$

The in-depth experimental characterization of Cu-ZnZrO$_x$ system under reaction conditions revealed the formation of low nuclearity zinc-rich Cu$_x$Zn$_y$ clusters stabilized on the ZrO_2 support, with Cu present in a metallic state and associated with partially reduced Zn atoms. We explored over 150 different structures to narrow down to the most representative model based on two criteria: (*i*) the compatibility with the experimental observations; and (*ii*) the DFT-computed energies were reasonable (*i.e.*, processes thermodynamically favored).

Firstly, we explored the incorporation and adsorption of Cu in the ZnZrO$_x$ model. While Cu incorporation (Cu@Zn$_4$ZrO$_x$) is unfavorable, its adsorption near Zn sites (Cu-Zn$_4$ZrO$_x$) is favored compared to zinc-free ZrO_2 (**Fig. 5**). Moreover, the adsorption of a second Cu atom is thermoneutral (Cu$_2$-Zn$_4$ZrO$_x$, **Fig. 5**), which is an indication of the Cu-Zn affinity, and can be linked to the computed exothermic formation energy of Cu_5Zn_8 and CuZn (E_{Cu5Zn8} = –0.26 eV and E_{CuZn} = –0.17 eV, **Supplementary Equations S5-S6**). Nevertheless, the Cu-Zn$_4$ZrO$_x$ model with isolated Zn atoms does not agree with the EXAFS findings, which suggest that Cu is most likely surrounded by a zinc-rich ensemble under reaction conditions (**Supplementary Table 4**). For this reason, we assessed the incorporation of *n* Zn atoms (*n* = 2-6) accompanied by *n* oxygen vacancies in nearby sites, as well as

the adsorption of one or two Cu atoms (**Supplementary Fig. 20-21** and **Supplementary Table 5**). The adsorption of Cu on these Zn-containing structures is also favored over zinc-free ZrO_2, further corroborating the Cu-Zn affinity.

The adsorption of a single Cu atom is particularly favored on the structure with 5 Zn and 5 oxygen vacancies, and the aggregation of a second Cu atom is exothermic by 0.50 eV, resulting in a particularly CuZn pattern. Notably, this structure is very similar to that exhibited by the one of the Cu_5Zn_8(110) surface (**Fig. 5**). Therefore, the Cu_2-Zn_5ZrO_x model was identified as the most stable among alternative models with 3, 4, or 6 Zn atoms. Since the latter fall within a range of 0.58 eV, the formation of other low-nuclearity zinc-rich clusters with catalytic properties similar to Cu_2-Zn_5ZrO_x cannot be fully excluded. We also assessed the formation of larger Cu clusters (containing 3 or 4 atoms) which are less stable from the thermodynamic standpoint than Cu_2-Zn_5ZrO_x structure (**Supplementary Figure 16**). Therefore, the latter was selected as the most representative model for the Cu-$ZnZrO_x$ catalyst.

To further ensure the robustness of the Cu_2-Zn_5ZrO_x model, we assessed alternative structures with distinct degrees of reduction. We computed 51 configurations representing the incorporation of two Zn atoms accompanied by two oxygen vacancies at different ZrO_2 surface sites (**Supplementary Fig. 17**). The potential energies of these structures were determined to fall within a range of 2.18 eV, indicating no correlation between the Zn-O_{vac} pair distance and its relative stability. Considering these results and the experimental findings, the Cu_2-Zn_5ZrO_x model is expected to contain five Zn-O_{vac} in close contact. To further confirm this hypothesis, we also examined similar structures containing 5 incorporated Zn atoms but with a lower degree of reduction (3 and 4 oxygen vacancies). The model comprising 5 oxygen vacancies is favored by more than 2.8 eV (**Supplementary Fig. 18**). Furthermore, we assessed the tendency of OH groups to fill oxygen vacancies located at the Cu_2-Zn_5ZrO_x ensemble. This process is thermoneutral (**Supplementary Fig. 19**), suggesting that some oxygen vacancies could potentially be filled by OH groups under reaction conditions. OH groups formed in this way will be easily removed and replenished during the process. Furthermore, the effect of potential OH groups would be negligible on the catalytic properties of Cu_2-Zn_5ZrO_x since the influence on the adsorption energies of CO_2, H_2, and CO was quantified to be less than 0.1 eV.

2.2. Additional insights into oxygen vacancy formation and dynamics

Fundamentally, each oxygen removed from the lattice releases two electrons into the conduction band that can be partially trapped in different sites such as empty vacancies and Zr^{4+} ions, which generate the two EPR-active signals (V_O^--p and Zr^{3+}). In contrast, exposure to the reaction mixture (CO_2+H_2) led to a decrease in the Zr^{3+} signal (**Fig. 6a** and **Supplementary Fig**. **22a**). This is consistent with previous observations indicating that Zr^{3+} is reoxidized to Zr^{4+} as electrons are transferred to vacancies (V_O^{2-}) formed by reaction with H_2.[1] Moreover, oxygen released from the lattice can also get reduced and stabilized on the surface as a superoxide ion (O_2^-), which is subsequently substituted by CO_2 and the electrons get transferred to the vacancies. Upon switching to reaction mixture, a newly broad signal attributed to an increase in the number of oxygen vacancies, the electrons stored in these defects start interacting ferromagnetically (exchange-coupled) (V_O^--f)[8] (**Fig. 6a** and **Supplementary Fig**. **22a**). At high vacancy concentrations, electrons are highly delocalized in V_O^--f, and thus the spin density per vacancy can be low. These V_O^--f signals are better fingerprints compared to V_O^--p for monitoring vacancy formation and annihilation. This signal is therefore more representative of the overall number of vacancies, particularly in comparison to the V_O^--p signal, which only takes the singly-filled and less reactive isolated vacancies likely structural (i.e. associated to charge balance when replacing Zr atoms by Zn) into account.

3. Supplementary Tables

Supplementary Table 1. Metal precursors used in the synthesis of the *M*-$ZnZrO_x$ catalysts.

Catalyst	Metal precursor[a]
Re-$ZnZrO_x$	$Re_2(CO)_{10}$
Co-$ZnZrO_x$	$Co(EHA)_2$
Au-$ZnZrO_x$	$AuCl_3$
Ni-$ZnZrO_x$	$Ni(acac)_2$
Rh-$ZnZrO_x$	$Rh(acac)_3$
Ag-$ZnZrO_x$	Ag(octanoate)
Ir-$ZnZrO_x$	$Ir(acac)_3$
Ru-$ZnZrO_x$	$Ru(acac)_3$
Pt-$ZnZrO_x$	$Pt(acac)_2$
Pd-$ZnZrO_x$	$Pd(acac)_2$
Cu-$ZnZrO_x$	$Cu(EHA)_2$
Cu-ZrO_x	$Cu(EHA)_2$
Cu-ZnO_x	$Cu(EHA)_2$

[a] All metal precursors were dissolved in a $Zn(EHA)_2/Zr(EHA)_4$ solution of the desired Zn/Zr = 5.0/94.5 ratio. The solvents EHA and THF were added to yield an overall metal concentration (*M*+Zn+Zr) of 0.40 mol kg^{-1} and a mass content of THF of 1/3 with respect to the total mass of the mixture.

Supplementary Table 2. Composition of the catalysts investigated.

Catalyst	Metal (mol%)		Zn (mol%)	
	Nominal	Measured[a]	Nominal	Measured[a]
$ZnZrO_x$	-	-	5	4.7
Re-$ZnZrO_x$	0.5	0.48	5	4.4
Co-$ZnZrO_x$	0.5	0.42	5	4.4
Au-$ZnZrO_x$	0.5	0.47	5	4.3
Ni-$ZnZrO_x$	0.5	0.54	5	4.5
Rh-$ZnZrO_x$	0.5	0.46	5	4.4
Ag-$ZnZrO_x$	0.5	0.46	5	4.5
Ir-$ZnZrO_x$	0.5	0.45	5	4.5
Ru-$ZnZrO_x$	0.5	0.52	5	4.4
Pt-$ZnZrO_x$	0.5	0.56	5	4.3
Pd-$ZnZrO_x$	0.5	0.49	5	4.3
Cu-$ZnZrO_x$	0.5	0.43	5	4.4
Cu-ZrO_x	0.5	0.51	-	-
Cu-ZnO_x	0.5	0.48	-	-

[a] ICP-OES.

Supplementary Table 3. Specific surface area, S_{BET} of the catalysts investigated in fresh form and after CO_2 hydrogenation. Reaction conditions as in caption of **Fig. 1**.

Catalyst	Status[a]	S_{BET}[b] ($m^2\ g^{-1}$)
$ZnZrO_x$	fresh	98
	20 h	97
	100 h	96
Re-$ZnZrO_x$	fresh	96
	20 h	98
Co-$ZnZrO_x$	fresh	103
	20 h	100
Au-$ZnZrO_x$	fresh	95
	20 h	101
Ni-$ZnZrO_x$	fresh	96
	20 h	96
Rh-$ZnZrO_x$	fresh	98
	20 h	101
Ag-$ZnZrO_x$	fresh	96
	20 h	96
Ir-$ZnZrO_x$	fresh	100
	20 h	100
Ru-$ZnZrO_x$	fresh	99
	20 h	101
Pt-$ZnZrO_x$	fresh	115
	20 h	99
Pd-$ZnZrO_x$	fresh	97
	20 h	98
Cu-$ZnZrO_x$	fresh	96
	20 h	99
	100 h	90
Cu-ZrO_x	fresh	103
	100 h	92
Cu-ZnO_x	fresh	61
	20 h	24

Supplementary Table 4. Results of the EXAFS spectra fitting of the catalysts presented in **Fig. 4c** and **Supplementary Fig. 8**.

Catalyst, status	Scattering path	Δ*E* (eV)	Number of neighbors	*R* (Å)	Debye-Waller factor ($Å^2$)
Cu-ZnZrO_x, fresh	Cu-O	7.8	3.3±0.1	1.94	0.002
	Zn-O	9.3	3.9±0.1	1.96	0.003
Cu-ZnZrO_x, CO_2+H_2, 300 min[a]	Cu-*M*	5.2	7.7±1.17	2.49	0.014
Cu-ZrO_x, fresh	Cu-O	8.8	3.5±0.1	1.93	0.004
Cu-ZrO_x, CO_2+H_2, 300 min[a]	Cu-Cu(Zn)	6.1	9.2±1.3	2.51	0.014
Pd-ZnZrO_x, used 20 h[b]	Pd-Zn	-4.3	5.8±0.6	2.59	0.008
	Pd-Pd	-4.3	2.93±0.1	2.95	0.013
	Pd-Pd	-4.3	1.47±0.1	3.43	0.013

[a] CO_2 hydrogenation conditions: m_{cat} = 0.013 g, F_T = 15 cm^3 min^{-1}, T = 573 K, P = 1.5 MPa, H_2/CO_2 = 4, dwell time = 300 min. [b] Reaction conditions as in caption of **Fig. 1**.

Supplementary Table 5. Potential energy, *E*, associated with different configurations of Cu_n-Zn_5ZrO_x obtained with **Supplementary Equations S1-S2** and associated with the models depicted in **Supplementary Fig. 20**. *n* (vertical) is the number of Cu atoms deposited on a *p*(2×2) slab of *m*-ZrO_2 (−111) surface with 5 Zn incorporated and 5 oxygen vacancies. The numbers in bold indicate the most stable configurations. The most stable configuration of each group with the same number of Cu atoms was employed as starting point to explore different configurations with an additional Cu atom.

n	*E*(eV)					
	1	2	3	4	5	6
0	**1.20**	1.48	1.81	2.72	1.64	1.54
1	3.16	2.83	3.38	3.13	3.59	**2.52**
2	3.55	3.46	3.55	3.56	3.00	**2.03**
3	3.91	**3.56**	4.13	3.94	4.90	4.56
4	4.45	**3.82**	4.60	4.45	5.01	5.02

Supplementary Table 6. Relative energy, Δ*E* between adsorbed formate and hydrogen (HCOO*+H*) and adsorbed methoxy and hydrogen (CH_3O*+H*) on different models. Δ*E* was obtained from the equation: $\Delta E = E^{\mathrm{DFT}}_{\mathrm{CH_3O*+H*}} + E^{\mathrm{DFT}}_{\mathrm{H_2O,gas}} - E^{\mathrm{DFT}}_{\mathrm{HCOO*+H*}} - 2E^{\mathrm{DFT}}_{\mathrm{H_2,gas}}$.

$\mathrm{HCOO^*+H^*+2H_{2,gas} \rightarrow CH_3O^*+H^*+H_2O_{gas}}$				
Model	*m*-ZrO_2(–111)	Cu(111)	$ZnZrO_x$	Cu_2-Zn_5ZrO_x
Δ*E* (eV)	–0.48	–0.73	–0.62	–0.76

Supplementary Table 7. DRIFTS band assignments of surface species detected on the catalysts presented in **Fig. 8a-f** and **Supplementary Fig. 28**.

Surface species	Wavenumber (cm^{-1})	Vibrational mode[a]
Formate ($HCOO^*$)[9–12]	1373	ν_s(OCO)
	1586	ν_{as}(OCO)
	2736	δ(CH)+ν_{as}(OCO)
	2874	ν(CH)
	2965	δ(CH)+ν_s(OCO)
Methoxy (CH_3O^*)[9–13]	1051	ν(CO)-terminal
	1140	ν(CO)-bridge
	2827	$\nu_s(CH_3)$
	2928	$\nu_{as}(CH_3)$

[a] ν_s and ν_{as} stand for symmetric and asymmetric vibrations, respectively.

Supplementary Table 8. Adsorption energy, E_{ads}, associated with different models shown in **Fig. 7** and acquired following the approach outlined in the **Computational details section**. Adsorption energy values obtained by considering dispersion and Hubbard corrections ($E_{ads,vdW}$ and $E_{ads,U}$, respectively) are shown for comparison.

Adsorbate		Model			
		m-ZrO_2 (–111)	Cu (111)	$ZnZrO_x$	Cu_2-Zn_5ZrO_x
CO_2	E_{ads}	–0.65	–0.01	–0.24	–0.85
	$E_{ads,vdW}$	–0.86	–0.23	–0.47	–0.89
	$E_{ads,U}$	–0.70	-	–0.30	–0.78
H_2	E_{ads}	0.34	–0.40	0.02	–0.77
	$E_{ads,vdW}$	0.19	–0.61	–0.09	–1.05
	$E_{ads,U}$	0.14	-	0.05	–0.73
CO	E_{ads}	–0.34	–0.86	–0.36	–0.69
	$E_{ads,vdW}$	–0.49	–1.07	–0.54	–0.90
	$E_{ads,U}$	–0.38	-	–0.35	–0.74
HCOO*	E_{ads}	–1.77	–0.48	–1.50	–1.73
	$E_{ads,vdW}$	–2.13	–0.93	–1.83	–2.10
	$E_{ads,U}$	–2.02	-	–1.78	–1.71
CH_3O*	E_{ads}	–2.25	–1.20	–2.12	–2.49
	$E_{ads,vdW}$	–2.60	–1.68	–2.57	–2.83
	$E_{ads,U}$	–2.39	-	–2.32	–2.48

Supplementary Table 9. Comparison between adsorption energies, E_{ads}, acquired following the approach outlined in the **Computational details section** and those obtained with dispersion and Hubbard corrections **(Supplementary Table 8).** $\Delta E_{ads,vdW}$ was calculated as $E_{ads,vdW} - E_{ads}$, while $\Delta E_{ads,vdW}$ was obtained from $E_{ads,U} - E_{ads}$. The inclusion of dispersion corrections leads to a systematic overstabilization of adsorbates while significant differences were not found between the E_{ads} obtained with our computational approach and by adding the Hubbard correction. Therefore, the general trends when comparing the adsorption of the key molecules on the different models are independent of the computational approach.

Adsorbate		Model			
		m-ZrO_2 (–111)	Cu (111)	$ZnZrO_x$	Cu_2-Zn_5ZrO_x
CO_2	$\Delta E_{ads,vdW}$	–0.21	–0.22	–0.23	–0.04
	$\Delta E_{ads,U}$	–0.05	-	–0.06	0.07
H_2	$\Delta E_{ads,vdW}$	–0.15	–0.22	–0.11	–0.29
	$\Delta E_{ads,U}$	–0.20	-	0.03	0.04
CO	$\Delta E_{ads,vdW}$	–0.15	–0.20	–0.18	–0.21
	$\Delta E_{ads,U}$	–0.04	-	0.01	–0.01
HCOO*	$\Delta E_{ads,vdW}$	–0.36	–0.45	–0.33	–0.37
	$\Delta E_{ads,U}$	–0.25	-	–0.28	0.02
CH_3O*	$\Delta E_{ads,vdW}$	–0.35	–0.47	–0.45	–0.34
	$\Delta E_{ads,U}$	–0.14	-	–0.20	0.02

4. Supplementary Figures

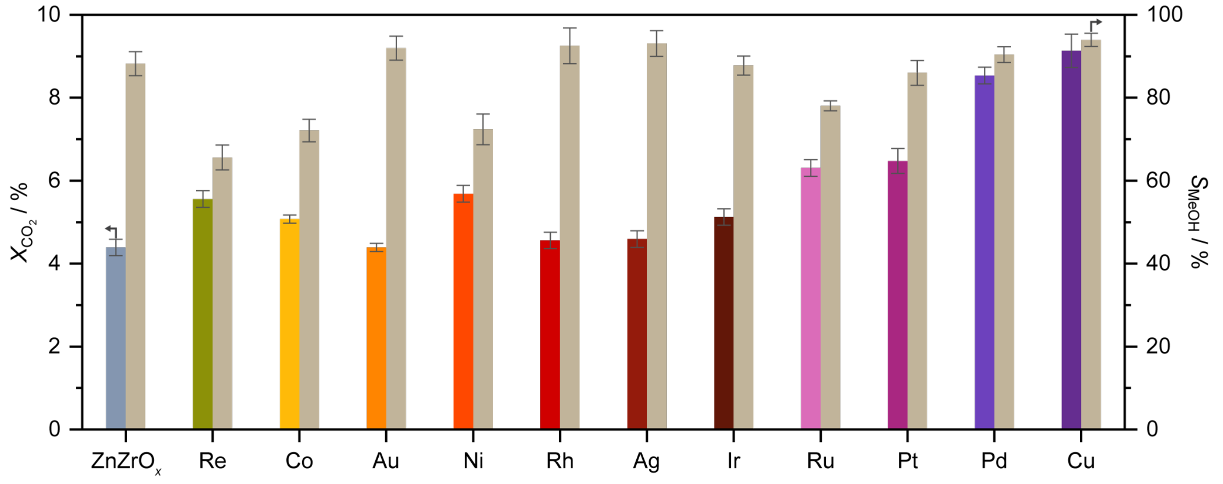

Supplementary Fig. 1. CO_2 conversion, X_{CO_2} and methanol selectivity, S_{MeOH} over ZnZrO$_x$ and *M*-ZnZrO$_x$ (0.5 mol% of metal, *M*) catalysts prepared by FSP. Reaction conditions as in caption of **Fig. 1**. Pd-ZnZrO$_x$ and Cu-ZnZrO$_x$ catalysts display higher CO_2 conversion while preserving high methanol selectivity, explaining their superior methanol space-time yield (**Fig. 1**) compared to the unpromoted ZnZrO$_x$ and other metal-containing systems.

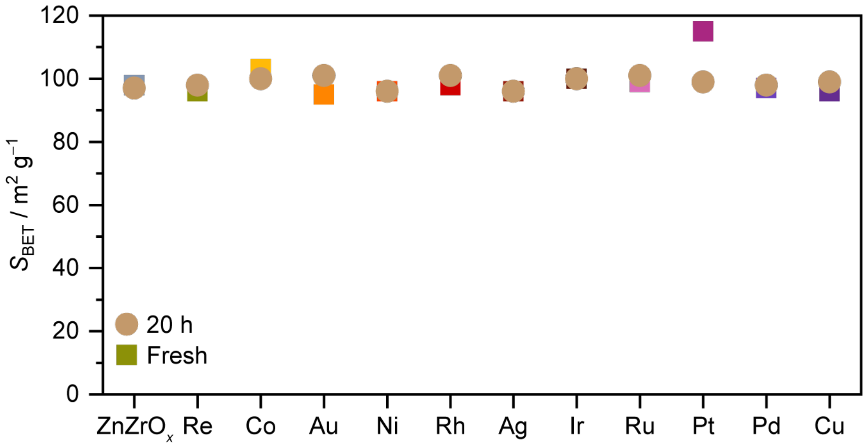

Supplementary Fig. 2. Specific surface area, S_{BET} of $ZnZrO_x$ and *M*-$ZnZrO_x$ catalysts in fresh form and after CO_2 hydrogenation for 20 h. Reaction conditions as in caption of **Fig. 1**. All catalysts display equivalent high S_{BET}, which is preserved upon reaction.

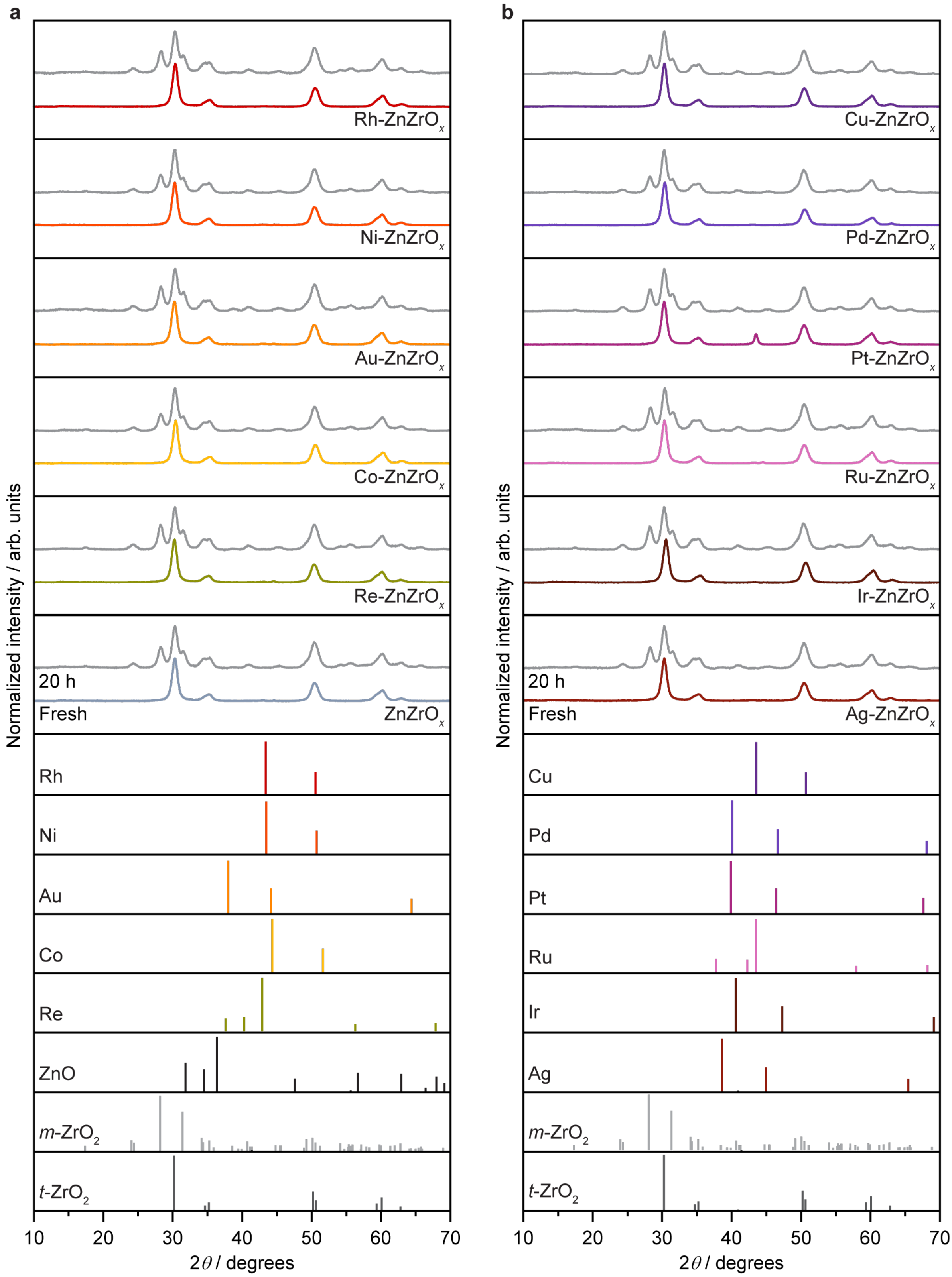

Supplementary Fig. 3. (**a**,**b**)XRD patterns of $ZnZrO_x$ and *M*-$ZnZrO_x$ catalysts in fresh form and after CO_2 hydrogenation for 20 h. Reaction conditions as in caption of **Fig. 1**. Reference patterns of pure phases are shown with vertical lines in the bottom panel. Upon reaction, the zirconia carrier transforms from a tetragonal to a monoclinic phase for all catalysts.

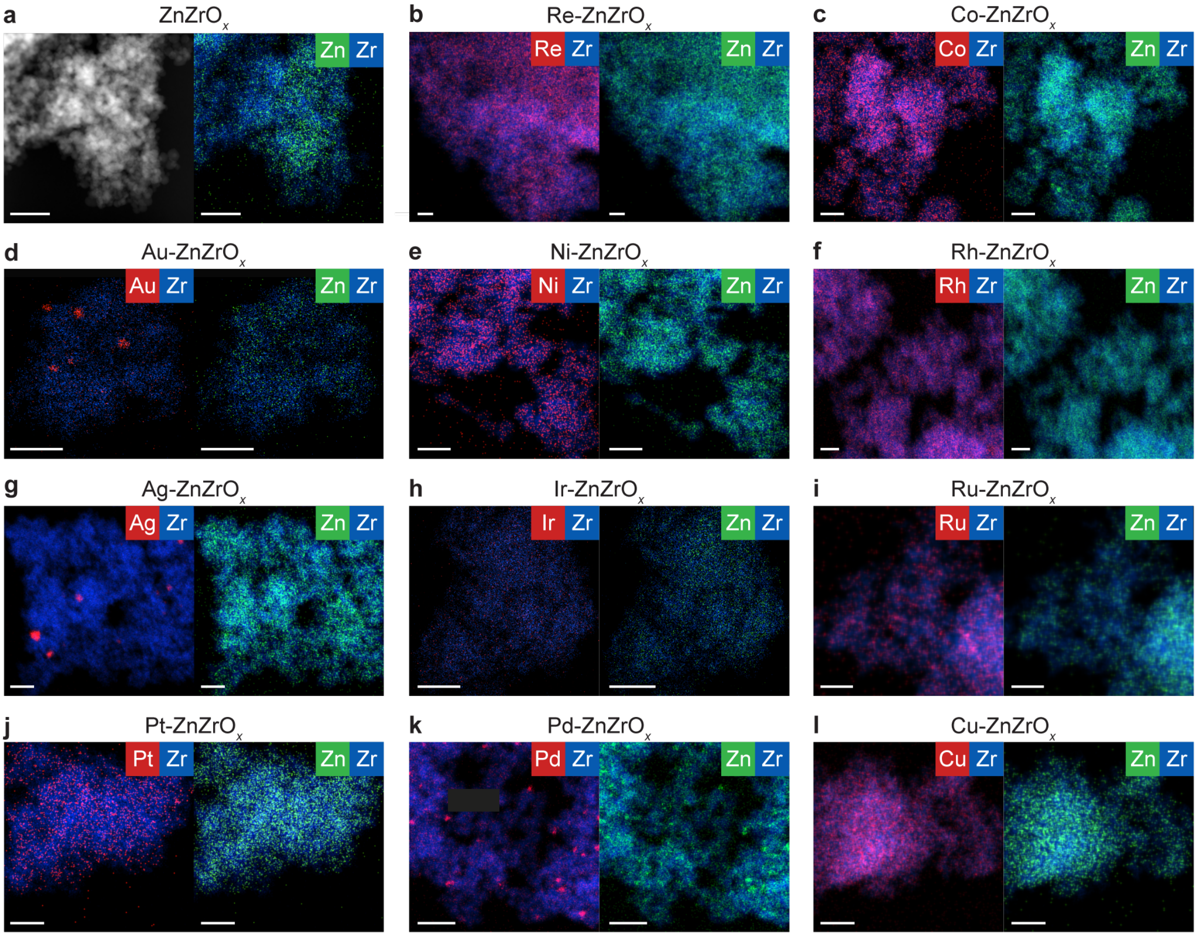

Supplementary Fig. 4. (**a**) HAADF-STEM micrograph and EDX map of $ZnZrO_x$ and (**b-l**) EDX maps of *M*-$ZnZrO_x$ catalysts after CO_2 hydrogenation for 20 h. Scale bars are equivalent to 50 nm. Reaction conditions as in caption of **Fig. 1**. While copper, rhodium, nickel, and rhenium form low-nuclearity species over $ZnZrO_x$, other metals are present as nanoparticles.

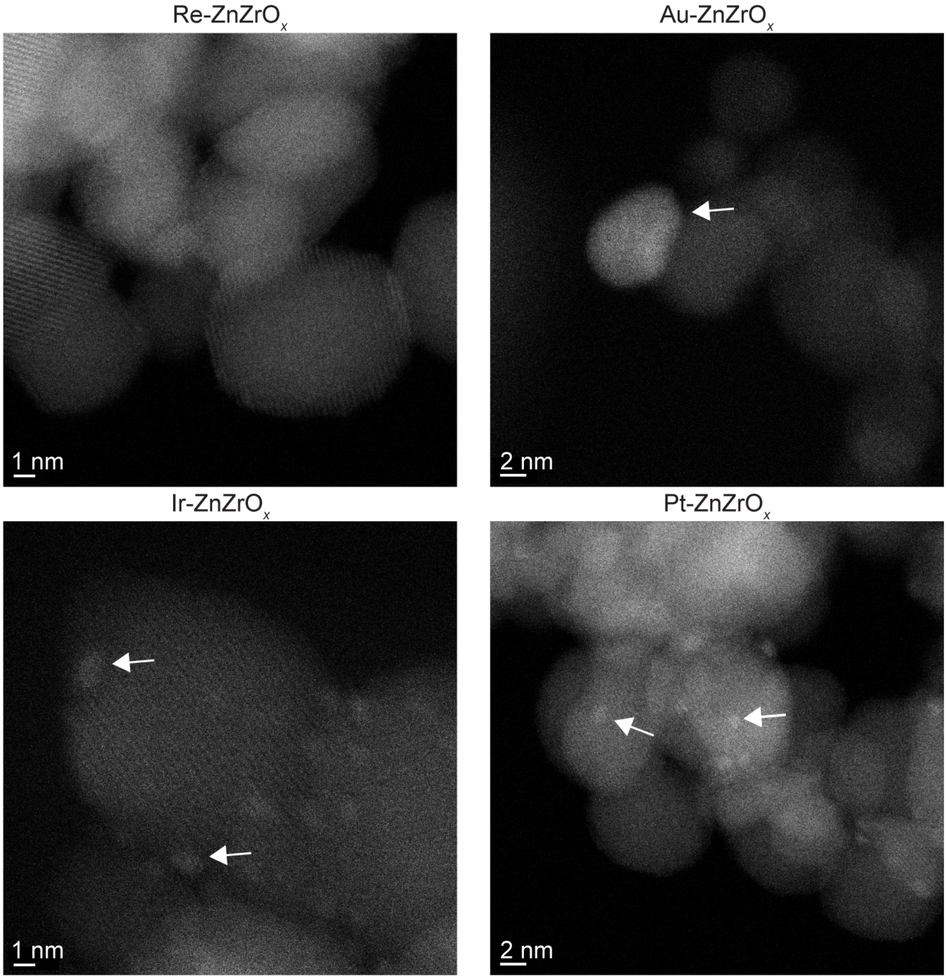

Supplementary Fig. 5. HAADF-STEM micrographs of selected *M*-ZnZrO$_x$ catalysts after CO_2 hydrogenation for 20 h. Reaction conditions as in caption of **Fig. 1**. White arrows indicate location of metal nanoparticles. While rhenium is atomically dispersed over Re-ZnZrO$_x$, nanoparticles of platinum (*ca.* 1 nm), iridium (*ca.* 1 nm), and gold (*ca.* 5 nm) are present over Pt-, Ir-, and Au-ZnZrO$_x$ catalysts, respectively.

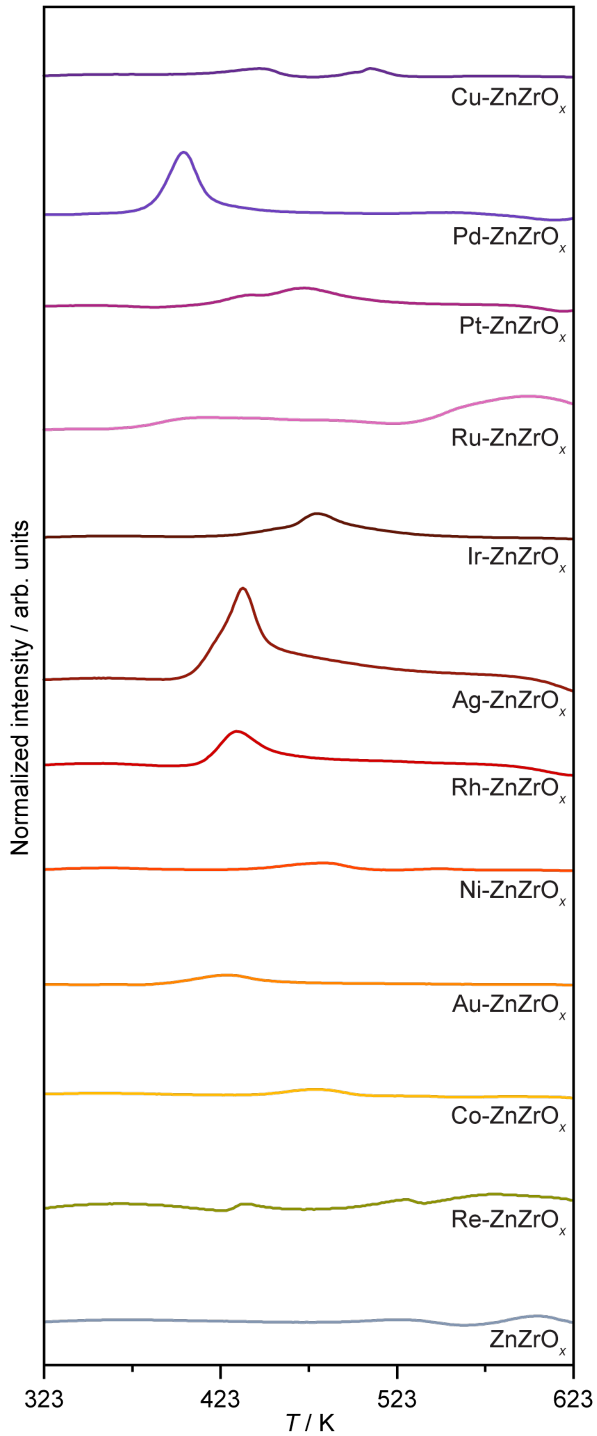

Supplementary Fig. 6. H_2-TPR profiles of *M*-ZnZrO_x catalysts. While no clear signals indicative of hydrogen consumption to form surface oxygen vacancies are observed for any of the catalysts, Pd-, Ir-, Ag-, and Rh-ZnZrO_x systems show distinctive features associated with the reduction of metal nanoparticles.

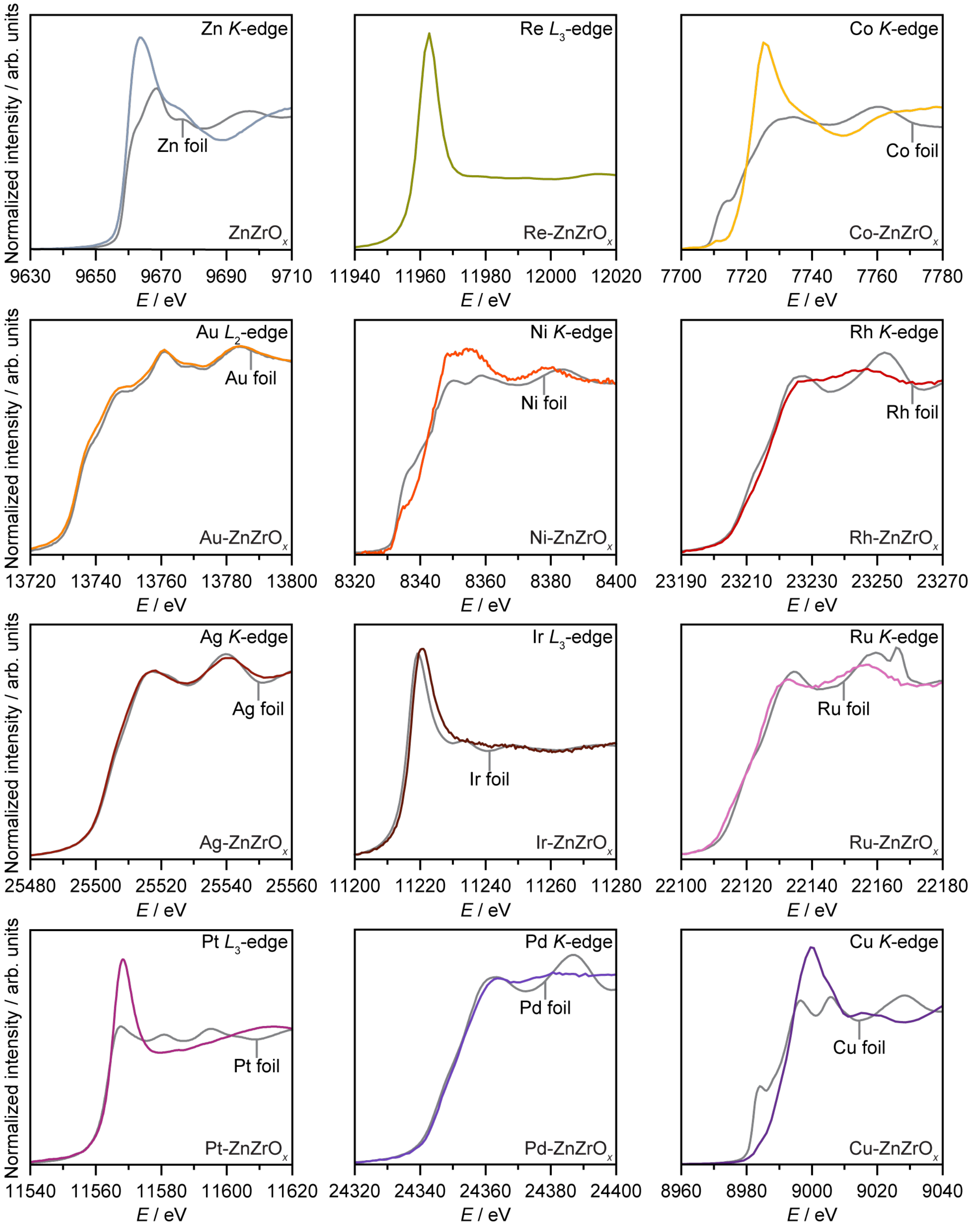

Supplementary Fig. 7. *k*-weighted metal edges XANES, with spectra of metallic phases serving as references, for the catalysts after activation in CO_2 hydrogenation for 20 h and preserved in inert atmosphere upon analysis. Reaction conditions as in caption of **Fig. 1**. Most metals are fully reduced, except rhenium, cobalt, and copper, which are oxidized.

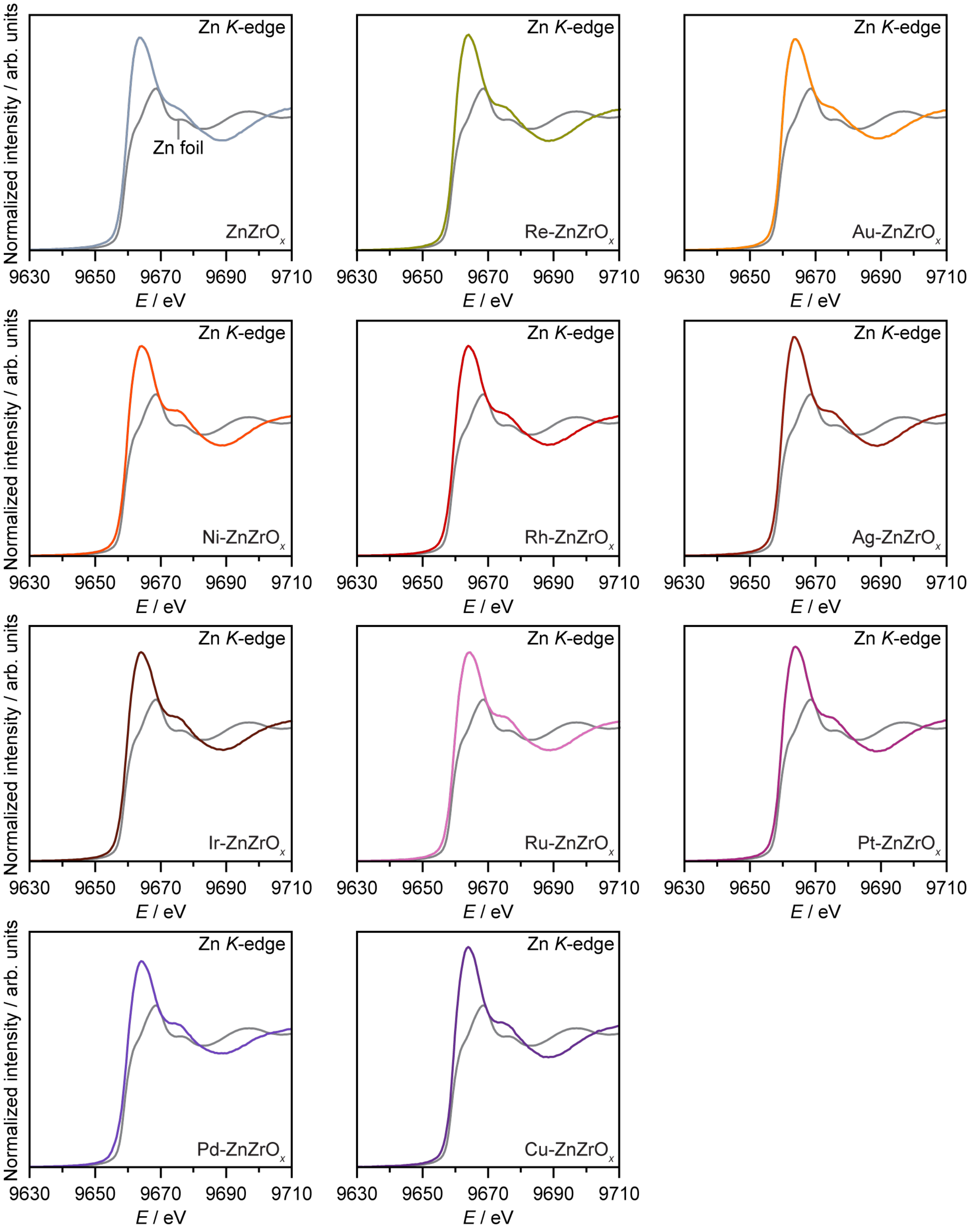

Supplementary Fig. 8. *k*-weighted Zn *K*-edge XANES, with spectra of metallic phases serving as references, for the catalysts after activation in CO_2 hydrogenation for 20 h and preserved in inert atmosphere upon analysis. Reaction conditions as in caption of **Fig. 1**. Zinc remains mostly oxidized for all catalysts, indicating that this speciation is predominant under reaction conditions.

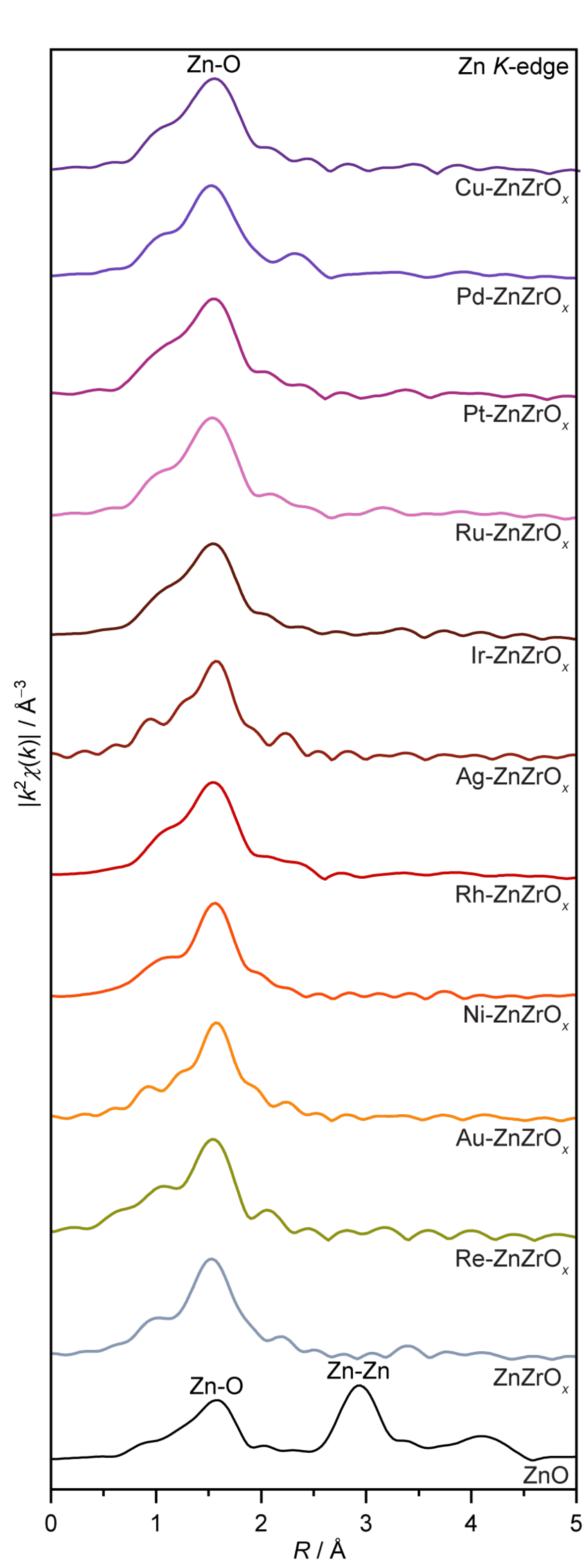

Supplementary Fig. 9. EXAFS spectra (Zn *K*-edge), with ZnO serving as reference, for *M*-ZnZrO$_x$ catalysts after activation in CO_2 hydrogenation for 20 h and preserved in inert atmosphere upon analysis. Reaction conditions as in caption of **Fig. 1**. Zinc remains oxidized and well dispersed upon reaction.

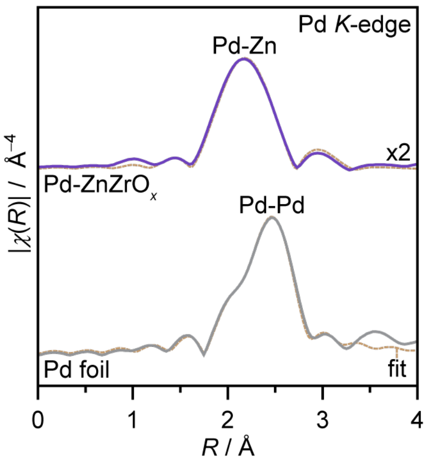

Supplementary Fig. 10. EXAFS spectra (Pd *K*-edge), with fit model and Pd foil serving as reference, for Pd-ZnZrO$_x$ catalyst after activation in CO_2 hydrogenation for 20 h and preserved in inert atmosphere upon analysis. Reaction conditions as in caption of **Fig. 1**. A palladium-zinc ally phase is formed over Pd-ZnZrO$_x$ catalyst upon reaction.

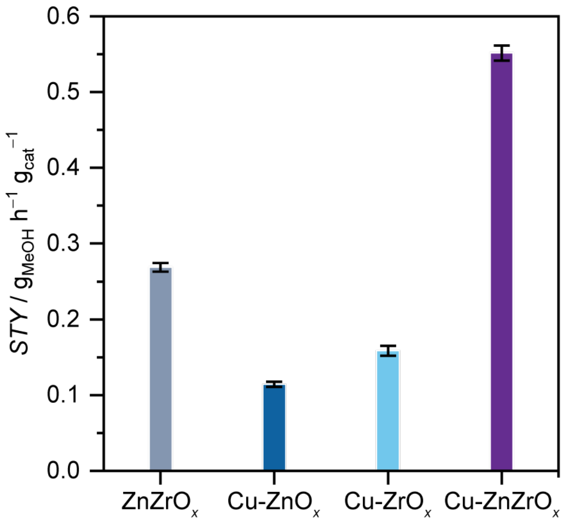

Supplementary Fig. 11. Methanol space-time yield, *STY* during CO_2 hydrogenation over $ZnZrO_x$, Cu-ZnO_x, Cu-ZrO_x, and Cu-$ZnZrO_x$ catalysts. Averaged values measured over 20 h on stream are presented with their corresponding error bars. Reaction conditions: $T = 573$ K, $P = 5$ MPa, $H_2/CO_2 = 4$, and $GHSV = 24{,}000$ cm^3 h^{-1} g_{cat}^{-1}. The reference $CuZnO_x$ catalyst display a performance comparable to Cu-ZrO_x but notably inferior to Cu-$ZnZrO_x$, further highlighting the superior catalytic properties of the ternary system.

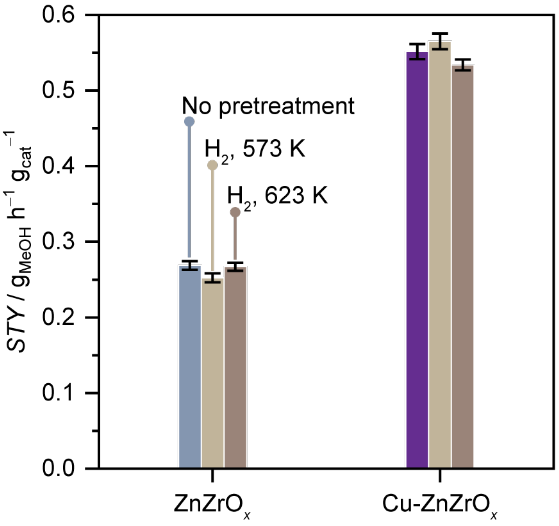

Supplementary Fig. 12. Methanol space-time yield, *STY* during CO_2 hydrogenation over $ZnZrO_x$ and Cu-$ZnZrO_x$ catalysts without (gray and purple bars) and with a pretreatment in H_2 at distinct temperatures (573 or 623 K) for 1 h. Averaged values measured over 20 h on stream are presented with their corresponding error bars. Reaction conditions: $T = 573$ K, $P = 5$ MPa, $H_2/CO_2 = 4$, and $GHSV = 24{,}000$ cm^3 h^{-1} g_{cat}^{-1}. No differences in methanol productivity were observed, indicating that pretreatment of the catalyst in H_2 likely does not influence the formation of oxygen vacancies. While other temperatures could be explored, temperatures higher than 623 K will likely induce sintering of the metal promoter, leading to detrimental effects on performance.

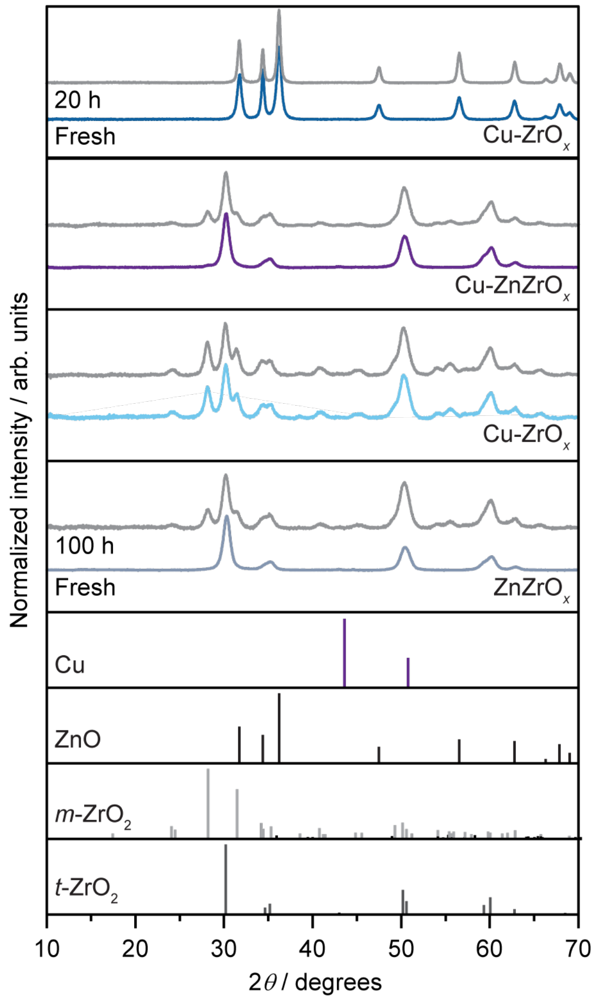

Supplementary Fig. 13. XRD patterns of ZnZrO$_x$, Cu-ZnO$_x$, Cu-ZrO$_x$, and Cu-ZnZrO$_x$ catalysts in fresh form and after CO_2 hydrogenation for 100 h (or 20 h). Reaction conditions as in caption of **Fig. 1**. Reference patterns of pure phases are shown with vertical lines in the bottom panel. No characteristic signal of copper and/or zinc oxide agglomeration is observed upon reaction, in line with their stable performance (**Fig. 3**).

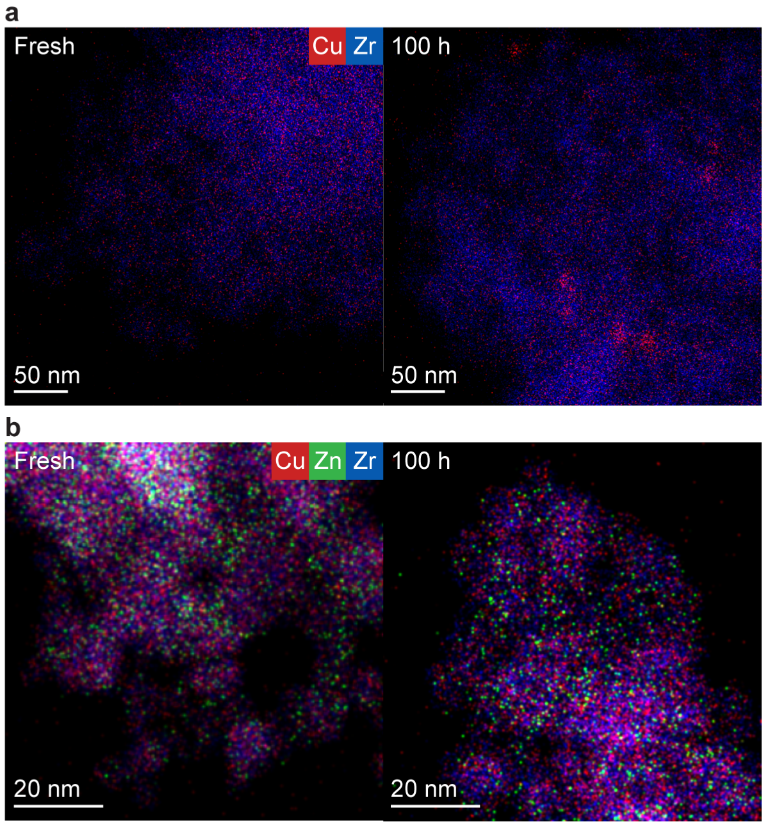

Supplementary Fig. 14. EDX maps of (**a**) Cu-ZrO_x, and (**b**) Cu-ZnZrO_x catalysts in fresh form and after CO_2 hydrogenation for 100 h. Reaction conditions as in caption of **Fig. 1**. While copper agglomerates into large nanoparticles (*ca.* 5nm) over Cu-ZrO_x upon reaction, both copper and zinc remain well dispersed over Cu-ZnZrO_x, explaining with the superior performance of the latter (**Fig. 3**).

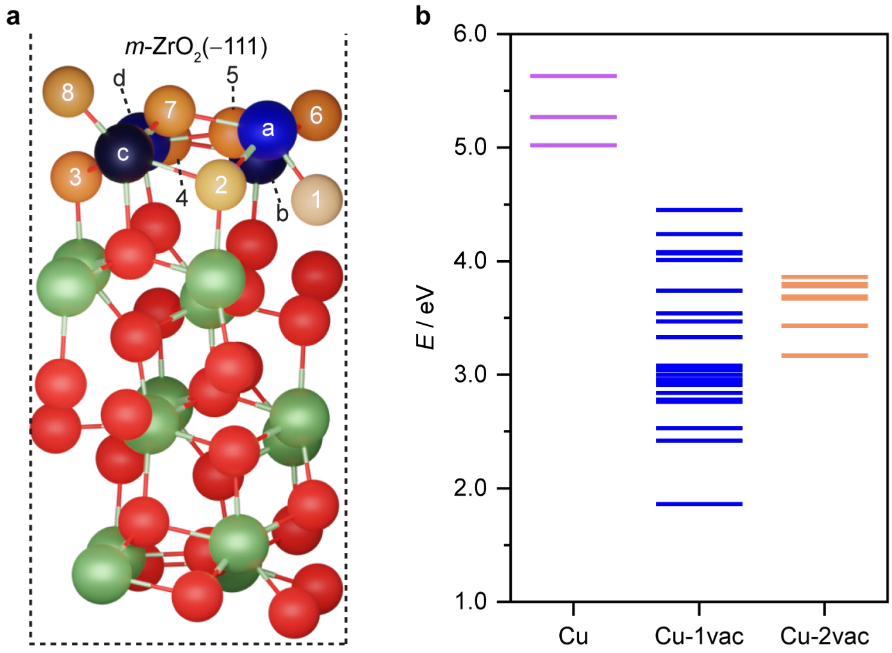

Supplementary Fig. 15. (**a**) Side view of the $p(1\times1)$ m-$ZrO_2(-111)$ slab model used to assess the Cu incorporation at different sites and the formation of oxygen vacancies. The four non-equivalent Zr surface sites (a-d) and the 8 non-equivalent O sites (1-8) are depicted in different shades of blue and orange, respectively. The other Zr and O atoms in the slab are colored in green and red, respectively. (**b**) Potential energy (E) associated with the incorporation of one Cu atom (Cu), the incorporation of one Cu and one oxygen vacancy (Cu-1vac), and the incorporation of one Cu and 2 oxygen vacancies (2-vac). The formation of the 1st oxygen vacancy was explored in all O non-equivalent sites with Cu incorporated to the 4 non-equivalent Zr sites. The formation of the 2nd oxygen vacancy was assessed by starting from the most stable configuration with one oxygen vacancy and considering the oxygen vacancy in the other seven remaining oxygens of the surface. All potential energies were obtained with the following equation, which is analog to **Supplementary Equations S1-S2** but i and v stands for the number of incorporated Cu and oxygen vacancies, respectively:

$$E = E^{\mathrm{DFT}}_{\mathrm{Cu}_i\mathrm{Zr}_{x-i}\mathrm{O}_{y-v}} + iE^{\mathrm{DFT}}_{\mathrm{ZrO}_{2,\mathrm{bulk}}} + (-2i+v)E^{\mathrm{DFT}}_{\mathrm{H_2O,gas}} - E^{\mathrm{DFT}}_{\mathrm{Zr}_x\mathrm{O}_y} - iE^{\mathrm{DFT}}_{\mathrm{Cu,bulk}} - (-2i+v)E^{\mathrm{DFT}}_{\mathrm{H_2,gas}} \,.$$

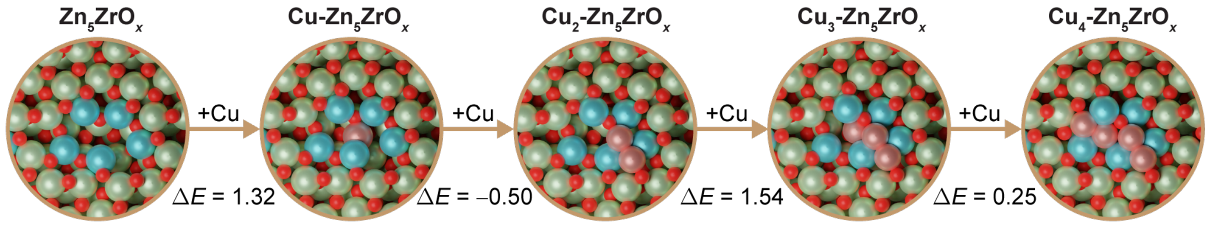

Supplementary Fig. 16. Relative energy (ΔE in eV) between Zn_5ZrO_x models with different amounts of deposited Cu atoms derived from **Supplementary Equations S1-S2**. Color code: Zr (green), Zn (blue), Cu (light pink), and O (red).

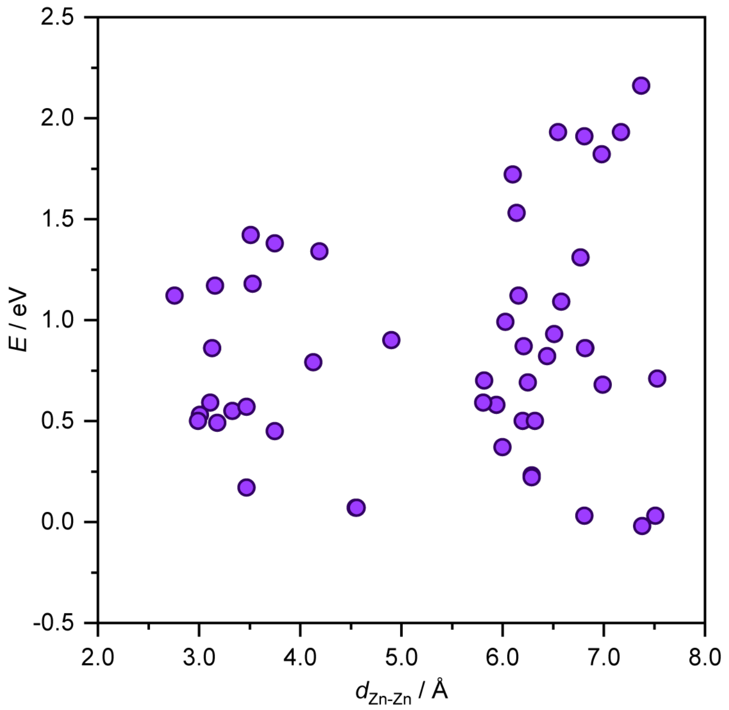

Supplementary Fig. 17. Potential energy, *E*, associated with models containing two Zn atoms incorporated into distinct sites of the ZrO_2 lattice accompanied by two oxygen vacancies as a function of the interatomic distance between Zn pairs.

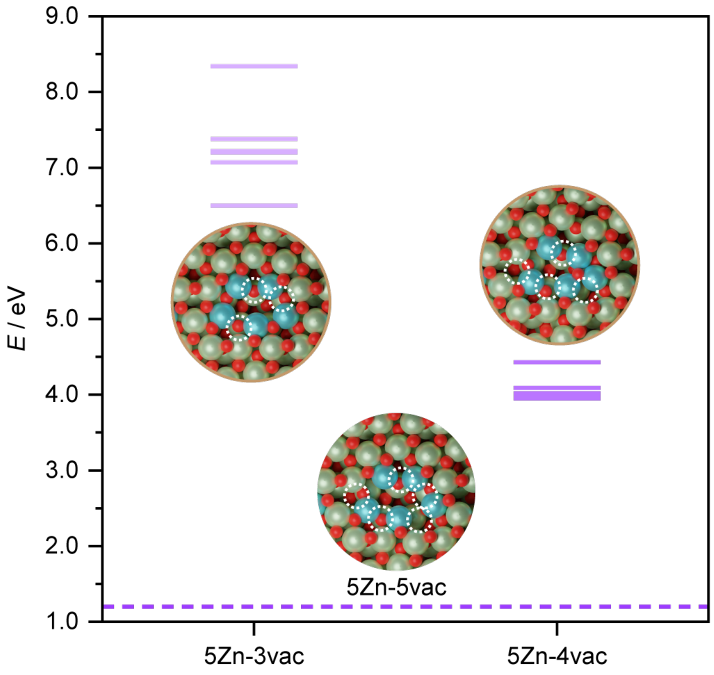

Supplementary Fig. 18. Potential energies, *E*, associated with structures incorporating five Zn atoms into the ZrO_2 lattice and different number of oxygen vacancies. The potential energy for the model with 5 Zn atoms and 5 oxygen vacancies (dashed line) is added for comaprison. Snapshots of the most stable models for each amount of oxygen vacancies. Color code: Zr (green), Zn (blue), Cu (light pink), O (red), and oxygen vacancy (dashed white).

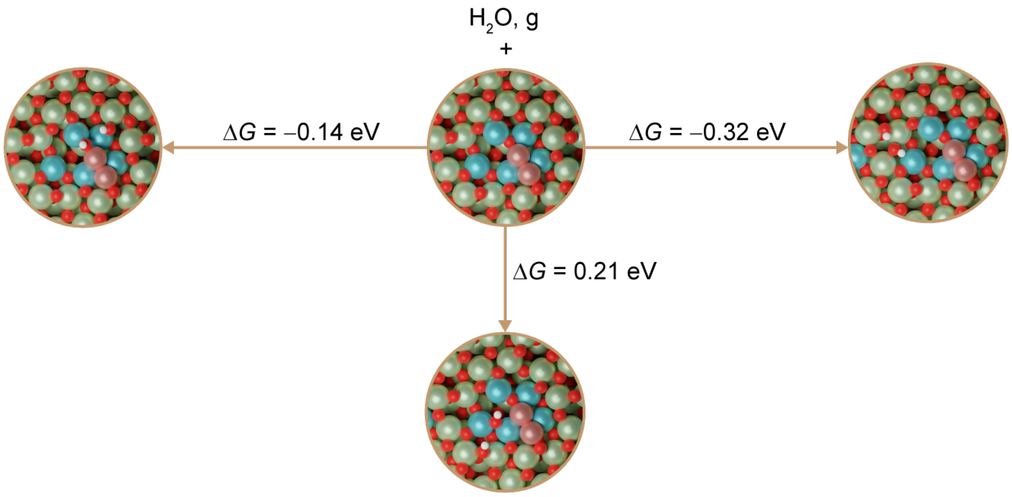

Supplementary Fig. 19. Snapshots and Gibbs free energy (ΔG) associated with the adsorption and dissociation of a water molecule from the gas phase on Cu_2-Zn_5ZrO_x at 573 K by healing an oxygen vacancy of the surface and forming a nearby OH. The contribution of vibrational modes to the enthalpy and entropy of the models with OH filled vacancies were computed to obtain the Gibbs free energies. Rotational and translational contributions were calculated for the molecule of water in gas-phase. Color code: Zr (green), Zn (blue), Cu (light pink), and O (red).

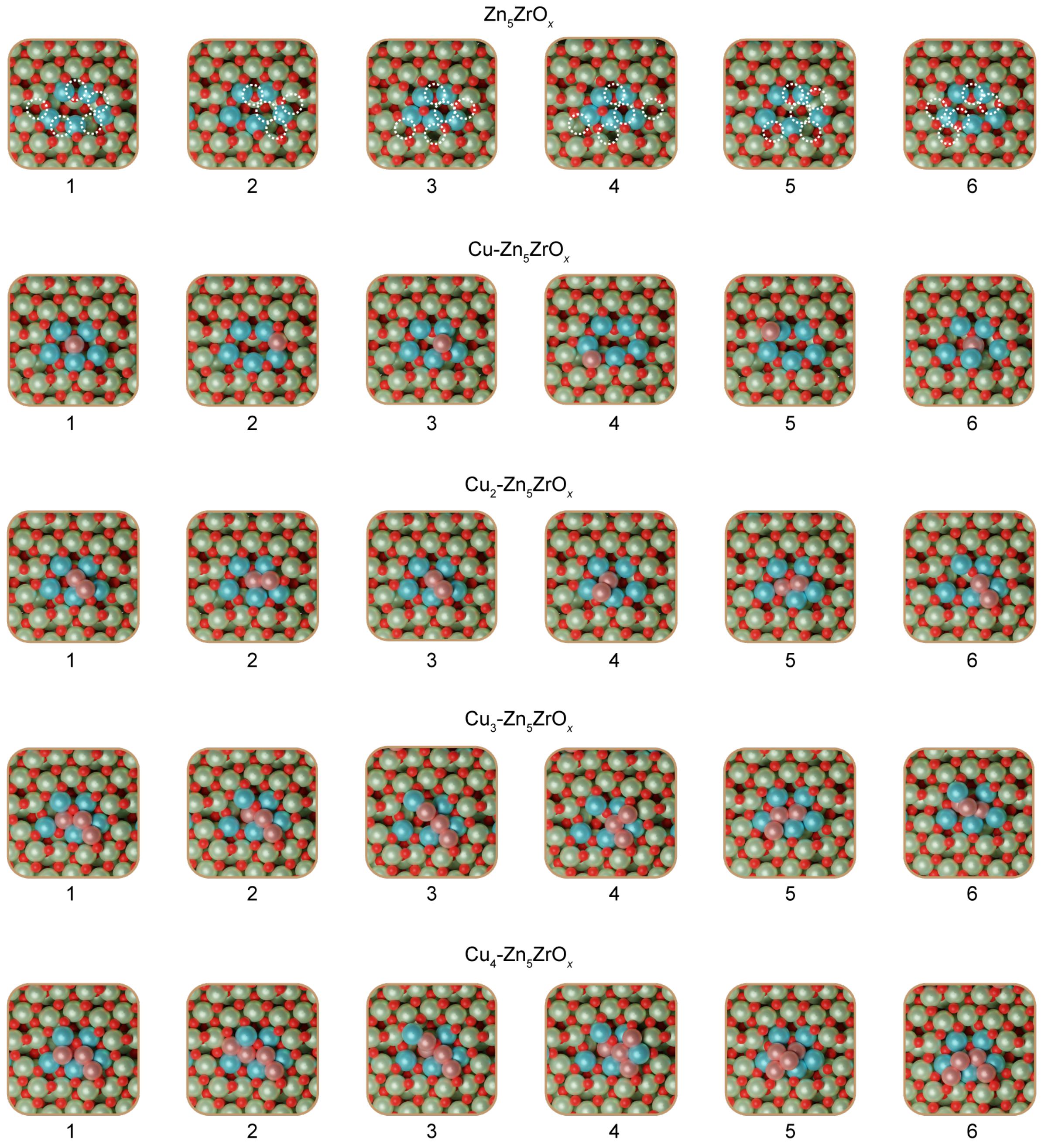

Supplementary Fig. 20. DFT models for different configurations of Cu_n-Zn_5ZrO_x associated with the potential energies provided in **Supplementary Table 6**. Color code: Zr (green), Zn (blue), Cu (light pink), O (red), and oxygen vacancy (dashed white).

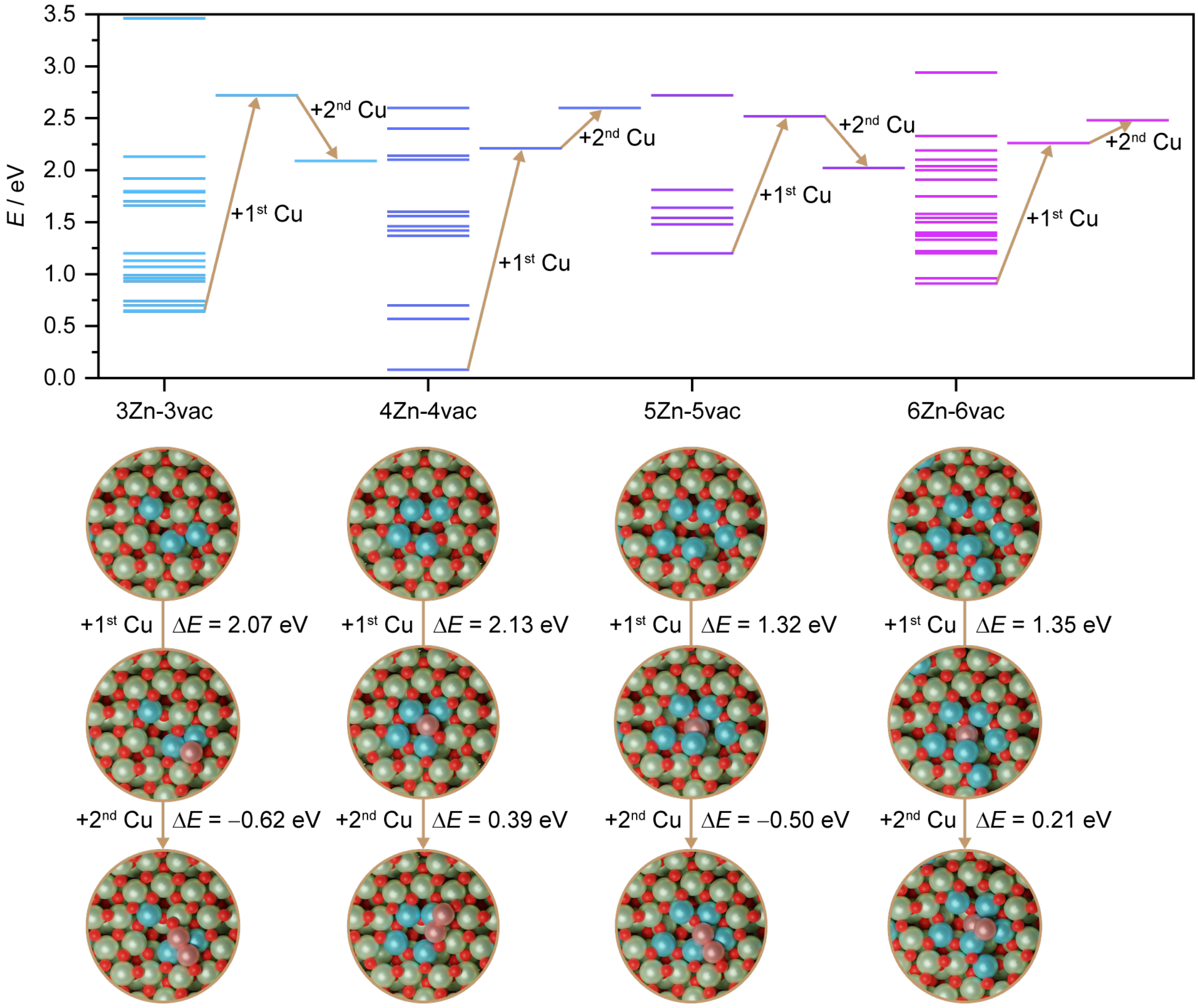

Supplementary Fig. 21. Potential energies, *E*, associated with structures with different number of Zn atoms incorporated into the ZrO_2 lattice, distinct number of oxygen vacancies, and considering the adsorption of one or two Cu atoms. The adsorption of Cu was explored by employing the most stable structure for each Cu-free Zn_nZnO_x (*n* = number of Zn atoms) model as the starting point. Snapshots of the most stable structures by means of *E* and the relative energy (Δ*E* in eV) between them are shown. Color code: Zr (green), Zn (blue), Cu (light pink), and O (red).

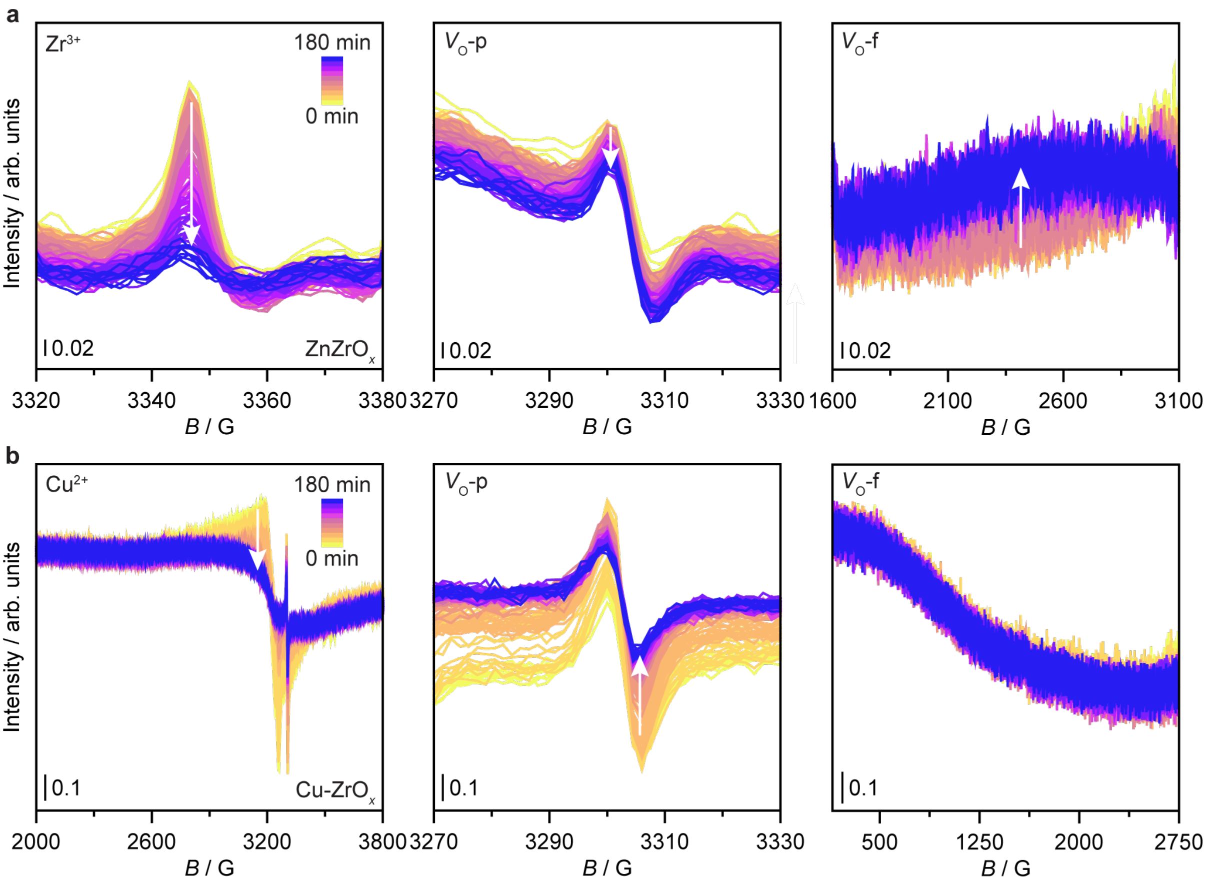

Supplementary Fig. 22. *Operando* EPR spectra of key signals for (**a**) $ZnZrO_x$ and (**b**) Cu-ZrO_x catalysts under He (m_{cat} = 0.013 g, F_T = 15 cm^3 min^{-1}, T = 573 K, P = 1 MPa, and dwell time = 30 min) and under reaction conditions (m_{cat} = 0.013 g, F_T = 15 cm^3 min^{-1}, T = 573 K, P = 1 MPa, and H_2/CO_2 = 4) with continuous time on stream. White arrows indicate trends of specific spectral features.

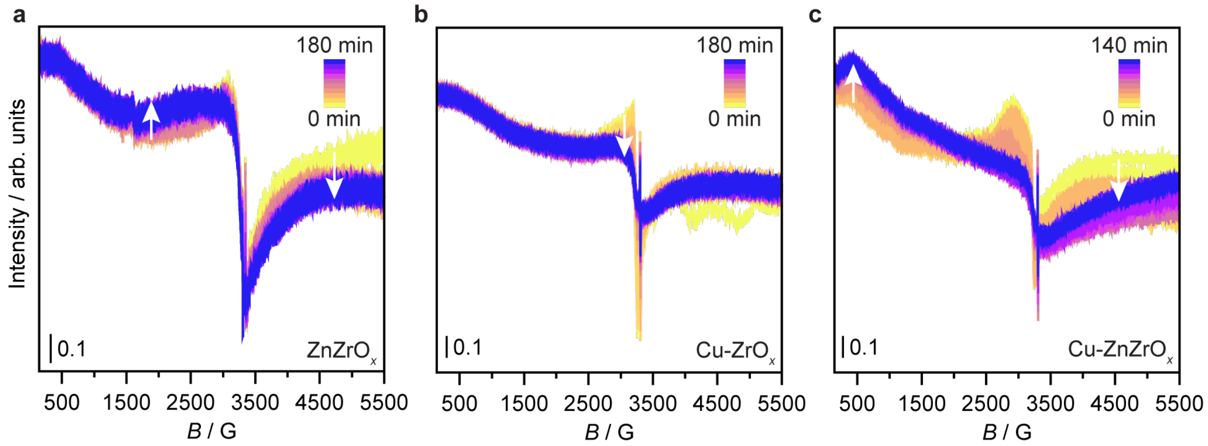

Supplementary Fig. 23. *Operando* EPR full spectra of (**a**) $ZnZrO_x$, (**b**) Cu-ZrO_x, and (**c**) Cu-$ZnZrO_x$ catalysts under He (m_{cat} = 0.013 g, F_T = 15 cm^3 min^{-1}, T = 573 K, P = 1 MPa, and dwell time = 30 min) and under reaction conditions (m_{cat} = 0.013 g, F_T = 15 cm^3 min^{-1}, T = 573 K, P = 1 MPa, and H_2/CO_2 = 4) with continuous time on stream. White arrows indicate trends of specific spectral features.

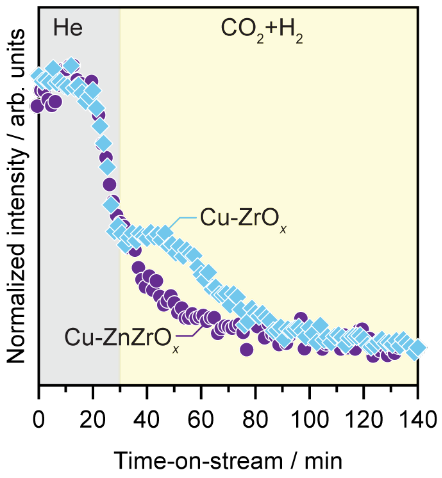

Supplementary Fig. 24. Evolution of the Cu^{2+} signal measured by *operando* EPR for Cu-ZrO$_x$ and Cu-ZnZrO$_x$ catalysts (**Fig. 6d** and **Supplementary Fig. 22b**) under under He (m_{cat} = 0.013 g, F_T = 15 cm^3 min^{-1}, T = 573 K, P = 1 MPa, and dwell time = 30 min) and under reaction conditions (m_{cat} = 0.013 g, F_T = 15 cm^3 min^{-1}, T = 573 K, P = 1 MPa, and H_2/CO_2 = 4) with continuous time-on-stream. The presence of zinc induces faster reduction of Cu^{2+} species over Cu-ZnZrO$_x$, and suggests coexistence of distinct copper speciation on Cu-ZrO$_x$.

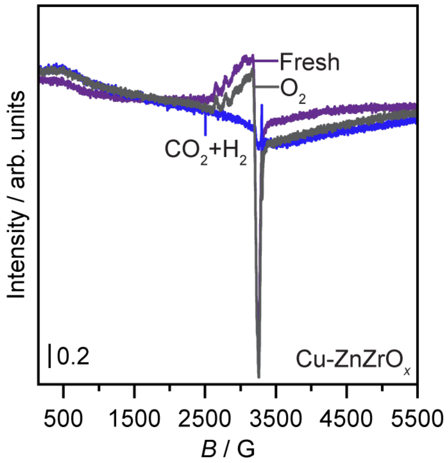

Supplementary Fig. 25. *In situ* EPR spectra measured at room temperature of Cu-ZnZrO$_x$ catalysts in fresh form, and CO_2 hydrogenation (m_{cat} = 0.013 g, F_T = 15 cm^3 min^{-1}, T = 573 K, P = 1 MPa, and H_2/CO_2 = 4), and after exposure to O_2 (m_{cat} = 0.013 g, F_T = 15 cm^3 min^{-1}, T = 313 K, P = 0.1 MPa). The Cu^{2+} signal is almost fully recovered with exposure to O_2 at room temperature, suggesting that only redox processes rather than copper aggregation events are linked to changes in the Cu^{2+} signal.

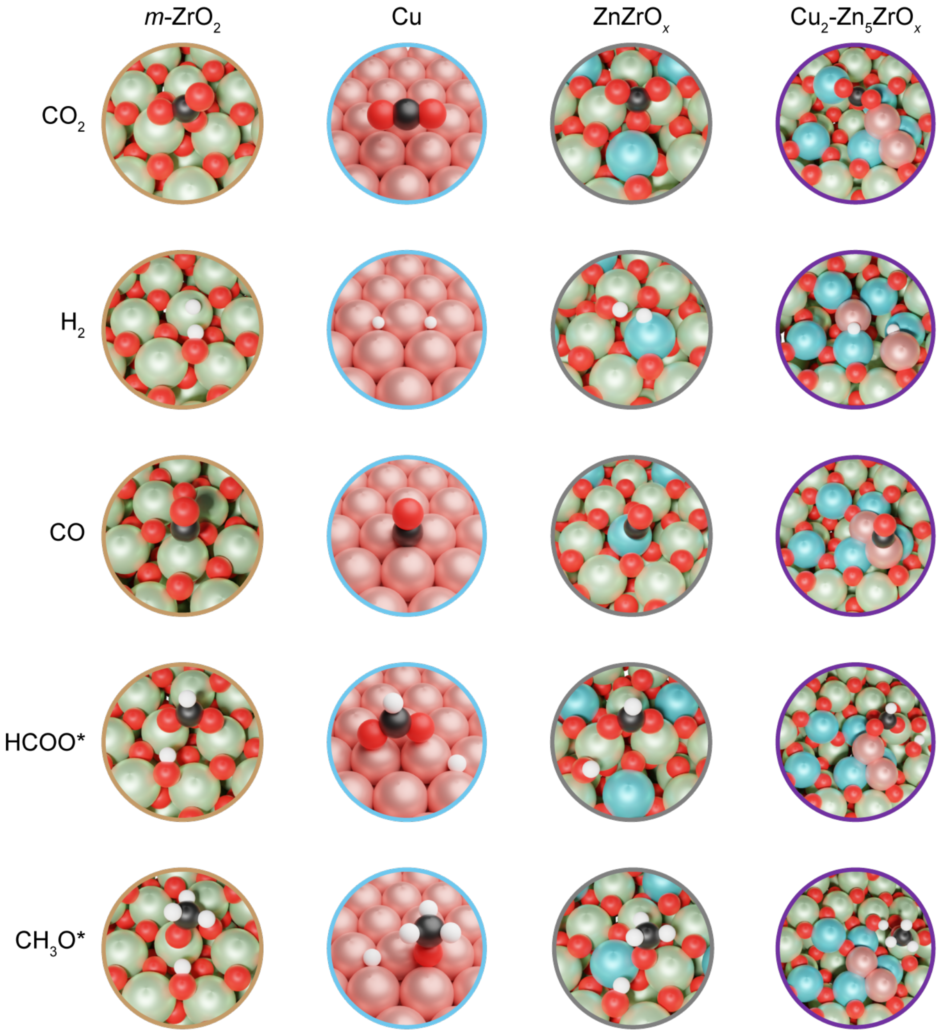

Supplementary Fig. 26. Snapshots illustrating the adsorption of CO_2, H_2, CO, HCOO*, and CH_3O* on the most relevant models associated with the adsorption energies depicted in **Fig. 7**. Color code: Zr (green), Zn (blue), Cu (light pink), and O (red).

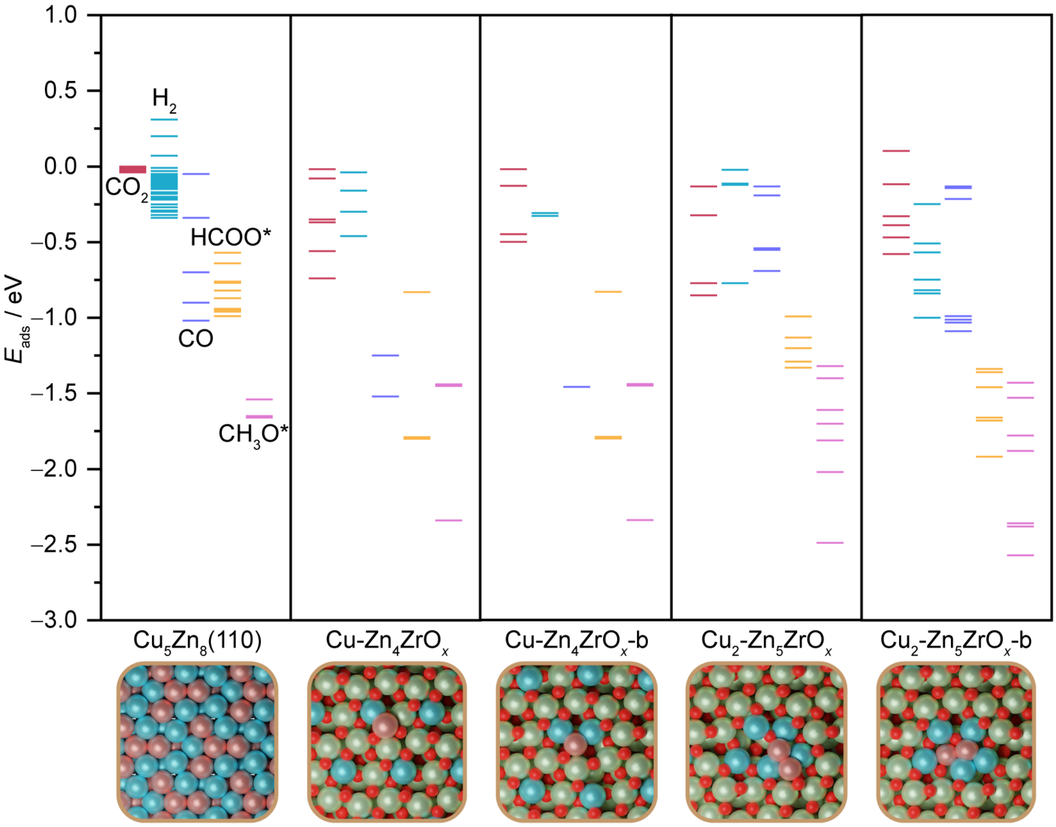

Supplementary Fig. 27. Adsorption energies, E_{ads} of reaction species on different models proposed to rationalize the performance of Cu-ZrO_x, $ZnZrO_x$, and Cu-$ZnZrO_x$ catalytic systems. Top views of the models employed in the DFT simulations are shown at t——he bottom panel. Color code of DFT models: Zr (green), Zn (blue), Cu (light pink), and O (red).

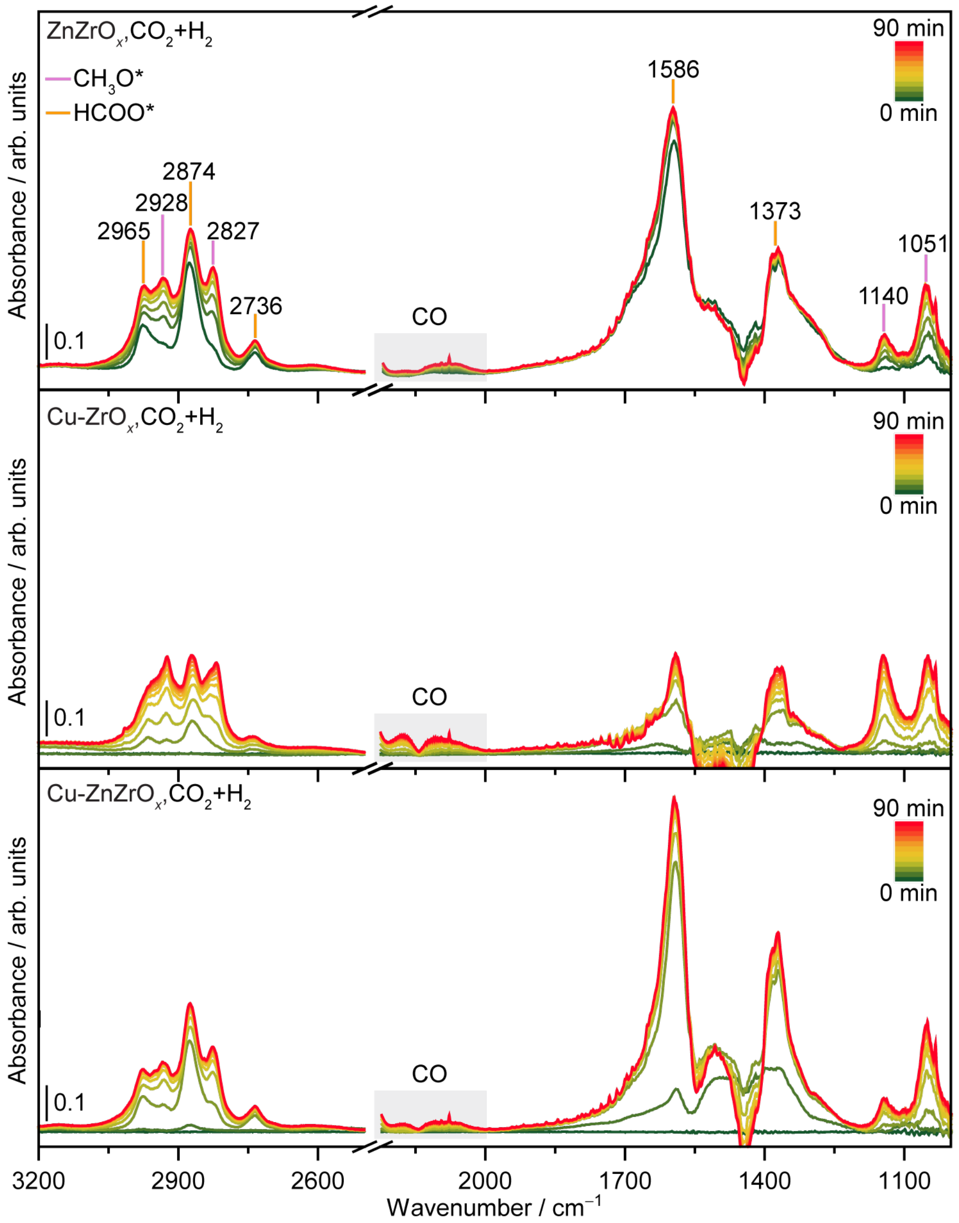

Supplementary Fig. 28. *Operando* DRIFT spectra for $ZnZrO_x$, $Cu\text{-}ZrO_x$, and $Cu\text{-}ZnZrO_x$ catalysts in CO_2 hydrogenation (F_T = 40 cm^3 min^{-1}, T = 573 K, P = 1.5 MPa, H_2/CO_2 = 4, and dwell time = 90 min).

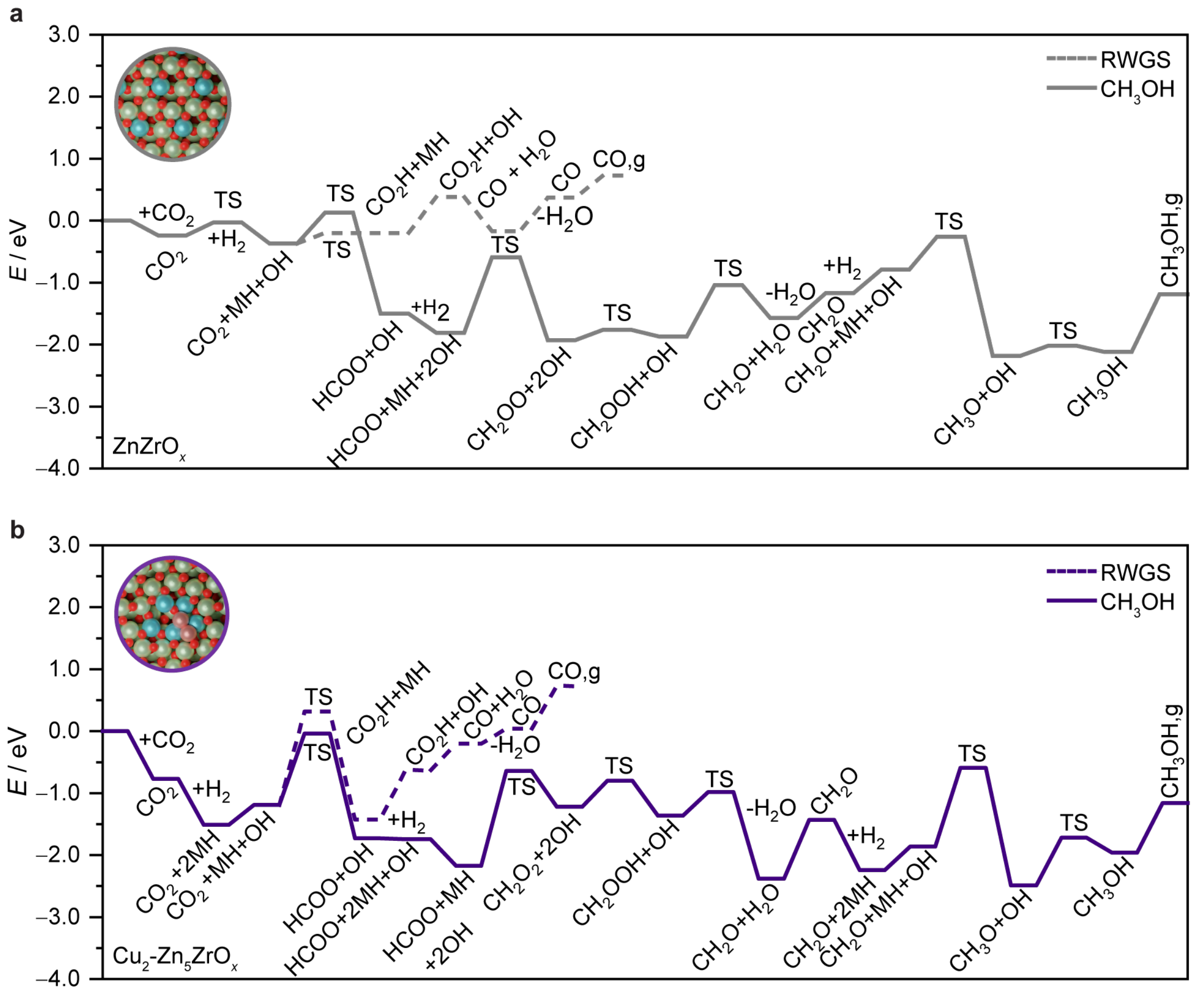

Supplementary Fig. 29. Energy profiles for CO_2 hydrogenation to CH_3OH (solid line) and the competing RWGS reaction (dashed line) on the (**a**) $ZnZrO_x$ and (**b**) Cu_2-Zn_5ZrO_x models. Color code of DFT models: Zr (green), Zn (blue), Cu (light pink), and O (red).

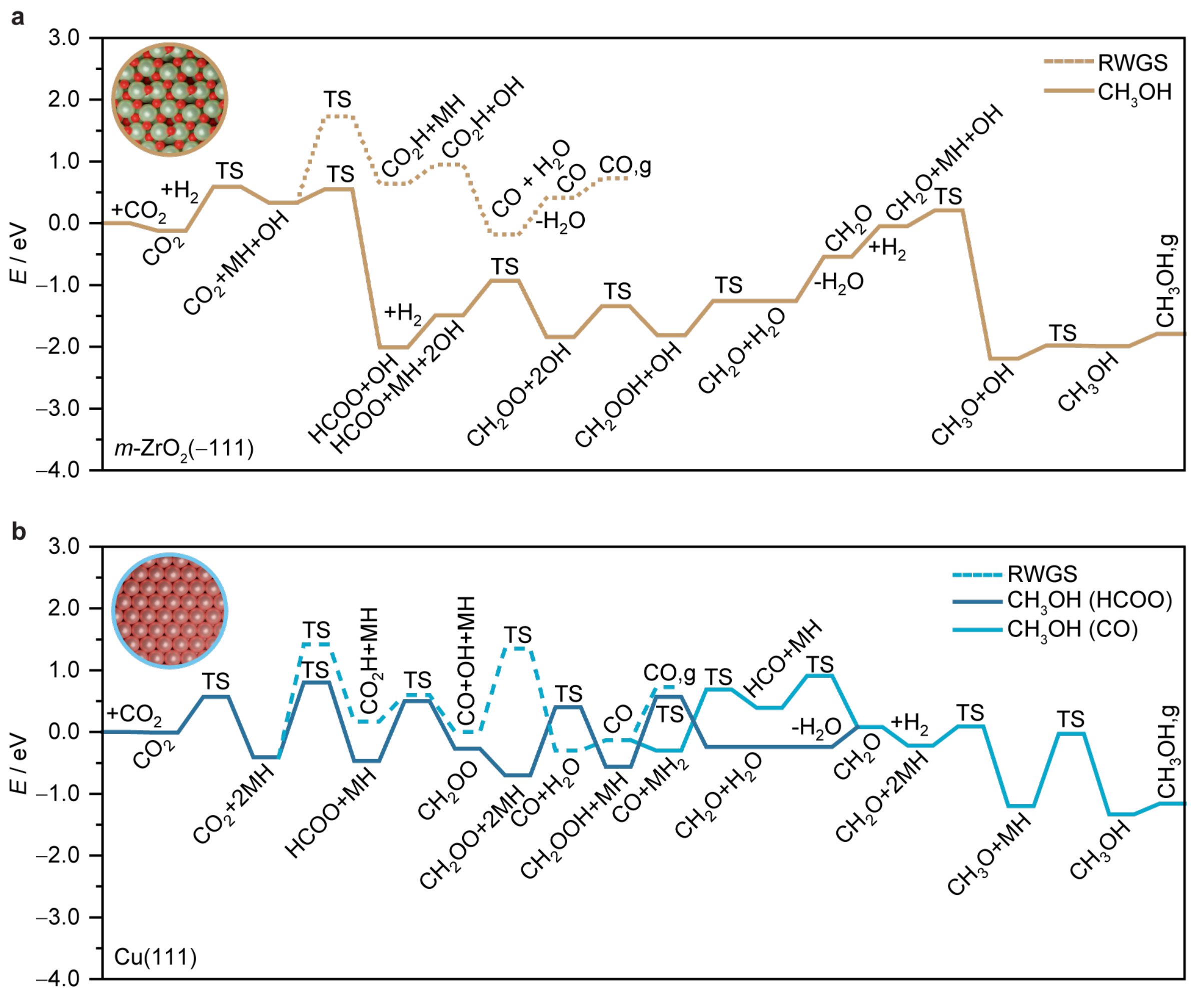

Supplementary Fig. 30. Energy profiles for CO_2 hydrogenation to CH_3OH (solid line) and the competing RWGS reaction (dashed line) on (**a**) *m*-ZrO_2(–111) and (**b**) Cu(111) models, which represent the Cu-ZnO_x catalyst. The formation of CH_3OH was evaluated through both the formate pathway (blue) and CO hydrogenation (dark blue) on Cu (111). Color code of DFT models: Zr (green), Zn (blue), Cu (light pink), and O (red).

5. Supplementary References

1. Pinheiro Araújo, T. *et al.* Design of flame-made $ZnZrO_x$ catalysts for sustainable methanol synthesis from CO_2. *Adv Ener. Mater.* **13**, 2204122 (2023).

2. Ruhle, M. Microscopy of Structural Ceramics. *Adv. Mater.* **9**, 195-217 (1991).

3. Taek Jung, K. & Bell, A. T. Effects of zirconia phase on the synthesis of methanol over zirconia-supported copper. *Catal. Letters* **80**, 63-68 (2002).

4. Lempelto, A., Gell, L., Kiljunen, T. & Honkala, K. Exploring CO_2 hydrogenation to methanol at a CuZn-ZrO_2 interface *via* DFT calculations. *Catal Sci Technol.* **13**, 4387-4399 (2023).

5. Arandia, A. *et al.* Effect of atomic layer deposited zinc promoter on the activity of copper-on-zirconia catalysts in the hydrogenation of carbon dioxide to methanol. *Appl Catal B* **321**, 122046 (2023).

6. Christensen, A. & Carter, E. A. First-principles study of the surfaces of zirconia. *Phys Rev B* **58**, 8050-8064 (1998).

7. Ricca, C., Ringuedé, A., Cassir, M., Adamo, C. & Labat, F. A comprehensive DFT investigation of bulk and low-index surfaces of ZrO_2 polymorphs. *J Comput. Chem.* **36**, 9-21 (2015).

8. Pinheiro Araújo, T. *et al.* Flame-made ternary Pd-In_2O_3-ZrO_2 catalyst with enhanced oxygen vacancy generation for CO_2 hydrogenation to methanol. *Nat Comm.* **13**, 5610 (2022).

9. Wang, J. *et al.* A highly selective and stable ZnO-ZrO_2 solid solution catalyst for CO_2 hydrogenation to methanol. *Sci. Adv.* **3**, 1-11 (2017).

10. Ma, Y. *et al.* Reactivity of a zirconia-copper inverse catalyst for CO_2 hydrogenation. *J. Phys. Chem. C* **124**, 22158-22172 (2020).

11. Wang, Y. *et al.* Strong Evidence of the Role of H_2O in Affecting Methanol Selectivity from CO_2 Hydrogenation over Cu-ZnO-ZrO_2. *Chem* **6**, 419-430 (2020).

12. Zhao, H. *et al.* The role of Cu_1–O_3 species in single-atom Cu/ZrO_2 catalyst for CO_2 hydrogenation. *Nat. Catal.* **5**, 818-831 (2022).

13. Yang, C. *et al.* Strong electronic oxide-support interaction over In_2O_3/ZrO_2 for highly selective CO_2 hydrogenation to methanol. *J Am Chem Soc* **142**, 19523-19531 (2020).
